# Bisphenol A induces otolith malformations during vertebrate embryogenesis

**DOI:** 10.1186/1471-213X-11-4

**Published:** 2011-01-26

**Authors:** Yann Gibert, Sana Sassi-Messai, Jean-Baptiste Fini, Laure Bernard, Daniel Zalko, Jean-Pierre Cravedi, Patrick Balaguer, Monika Andersson-Lendahl, Barbara Demeneix, Vincent Laudet

**Affiliations:** 1 Institut de Génomique Fonctionnelle de Lyon; Université de Lyon; Université Lyon 1; CNRS; INRA; Ecole Normale Supérieure de Lyon; 46 allée d'Italie, 69364 Lyon Cedex 07, France; 2 CNRS UMR 7221, Département Régulations, Développement et Diversité Moléculaire, Muséum National d'Histoire Naturelle, 7 rue Cuvier, 75231 Paris Cedex 5, France; 3 INSERM Equipe « Signalisation Hormonale, Environnement et Cancer ». Centre en recherche de cancérologie de Montpellier, Parc Euromédecine-CRLC Val d'Aurelle, F-34298 Montpellier Cedex 5, France; 4 INRA, UMR 1089 Xénobiotiques, 180 chemin de Tournefeuille, BP 93173, F-31300 Toulouse, France; 5 Karolinska Institutet, Department of Biosciences and Nutrition, Laboratory of Medical Nutrition, NOVUM KUS, SE-14186 Stockholm, Sweden

## Abstract

**Background:**

The plastic monomer and plasticizer bisphenol A (BPA), used for manufacturing polycarbonate plastic and epoxy resins, is produced at over 2.5 million metric tons per year. Concerns have been raised that BPA acts as an endocrine disruptor on both developmental and reproductive processes and a large body of evidence suggests that BPA interferes with estrogen and thyroid hormone signaling. Here, we investigated BPA effects during embryonic development using the zebrafish and *Xenopus *models.

**Results:**

We report that BPA exposure leads to severe malformations of the otic vesicle. In zebrafish and in *Xenopus *embryos, exposure to BPA during the first developmental day resulted in dose-dependent defects in otolith formation. Defects included aggregation, multiplication and occasionally failure to form otoliths. As no effects on otolith development were seen with exposure to micromolar concentrations of thyroid hormone, 17-ß-estradiol or of the estrogen receptor antagonist ICI 182,780 we conclude that the effects of BPA are independent of estrogen receptors or thyroid-hormone receptors. Na^+^/K^+ ^ATPases are crucial for otolith formation in zebrafish. Pharmacological inhibition of the major Na^+^/K^+ ^ATPase with ouabain can rescue the BPA-induced otolith phenotype.

**Conclusions:**

The data suggest that the spectrum of BPA action is wider than previously expected and argue for a systematic survey of the developmental effects of this endocrine disruptor.

## Background

Bisphenols represent a group of industrial chemicals, widely and abundantly used for the production of polycarbonate plastics and epoxy resins. The most common bisphenol, bisphenol A [2,2-bis(4-hydroxyphenyl)propane; BPA] is used in the manufacture of plastic wares, dental resins, food can lining and flame retardants. Total annual production of BPA in the world exceeds 2.5 million metric tons [1]. Bisphenols may be present in the environment as a result of direct release from manufacturing or processing facilities or release of unreacted monomers. According to the recent NTP report total environmental release of BPA in 2004 was 82 tons [2]. BPA is among the most frequent organic wastewater contaminants detected in ground water in the USA [3]. In the United States, the median reported BPA concentration in surface waters was 0.14 μg/L whereas in Europe, mean values of 0.0047 μg/L and 0.0052 μg/L were observed in Germany and Netherlands respectively [4-6]. However, BPA levels found in receiving waters near processing facilities can occasionally reach levels higher than 20 μg/L [4]. The presence of BPA in fish has been examined by several authors. Basheer et al. found that BPA residues in the fish *Decapterus russelli *from Singapore averaged 65.6 ng/g [7]. Concentrations ranging from 2 to 75 ng/g in the liver and from 1 to 11 ng/g in muscle were found in flounder (*Platichthys flesus*) and bream (*Abramis brama*) respectively. These fish were collected from Dutch coastal waters [4].

The highest potential for human exposure to BPA is through products that directly contact food like beverage containers with internal epoxy resin coatings, polycarbonate tableware and feeding bottles. Assessment of human BPA exposure by biomonitoring urinary excretion of BPA metabolites in the population gives an estimated average daily exposure of BPA up to 7 μg/adult/day and up to 66 μg/day in six months old infants (EFSA, 2006). Measurements of BPA in serum of adult men and women show mean concentrations of 1.49 and 0.64 ng/mL respectively [8,9]. Presence of BPA was also reported in maternal and fetal plasma, in placental tissue in humans [10,11] and in the milk of nursing mothers [12]. Most disquieting is the concentration of BPA in amniotic fluid that is approximately five-fold higher than levels measured in maternal plasma [10].

Several bisphenols have been shown to cause adverse effects in vertebrates, including humans, by interfering with normal endocrine function [13-16] and are thus classified as endocrine disruptors (EDs). The available data on the interactions between BPA and the endocrine systems suggest it could trigger a wide variety of adverse effects *in vivo*. BPA displays estrogenic activity in a number of experimental systems and thereby has the potential to adversely affect reproductive function and development in humans and wildlife [17-19]. It can bind to and act as a weak agonist on both estrogen receptors ERα and ERβ with affinity constants of 0.1 and 1 μM respectively [20-22] but can also be an antagonist in some type of cells via ERα [23]. Thus, it is defined as a selective ER modulator [24]. In addition, data from recent studies have revealed that low levels of BPA can induce rapid, membrane-initiated effects similar to the non-genomic effects induced by estrogen [25,26]. Thus, BPA exposure could result in interference with normal estrogenic signalling at multiple levels and at different developmental stages as well as in the adult. In addition, BPA has been shown to interact with other nuclear receptor systems such as the thyroid hormone (TH) receptors (TRs). BPA is a TR antagonist in transient transfection assays in mammalian cells and BPA exposure of rats during pregnancy causes an increase in serum thyroid hormone concentration [27-29]. BPA acts as a TR antagonist in a tail culture assay in *Xenopus *[30]. Further, BPA inhibits T3-dependent responses in an *in vivo Xenopus *embryo TH sensitive gene transcription reporter assay [31]. All these data suggest that BPA not only acts on the reproductive system via the estrogen receptors but can exert a wide range of actions through various nuclear receptor targets during development.

Little attention has been paid to the effects of BPA during early developmental stages. Ramakrishnan and Wayne showed that exposure to low levels of BPA accelerated early embryonic development within 24h of exposure, attenuated body growth and advanced the times of hatching and reproductive maturation in medaka fish (*Oryzias latipes*). It was also shown that high dose of BPA exposure led to zebrafish embryo mortality while sub-lethal doses led to no blood flow, cardiac edema and tail malformation [32]. In the medaka embryo, 200 microg BPA/l caused transient embryonic deformities in medaka between days 4 and 8. By day 9 BPA treated embryos were not different from untreated siblings [33].

This lack of focus on early development may be due in part to the fact that mammalian embryos are difficult to access experimentally. A few recent papers have used the free-living *Xenopus laevis *embryo to test the effects of BPA at different stages of development [34,35]. In particular, BPA was shown to cause malformations of the head region including the eye [36]. We too chose to exploit free-living embryos of *Xenopus *and zebrafish to address the possibility that BPA might affect early development and organogenesis. The advantage of using aquatic embryos is the ease in adding substances to be investigated to the aquarium water and study development following exposure without requiring the injection/dissection of the mother as in mammalian models. Furthermore, fish and amphibian embryos offer a unique combination of advantages for studying genetics and developmental biology during vertebrate organogenesis, as they are transparent, thereby allowing non-invasive observation. In addition, the large numbers of embryos per clutch provides large sample numbers. In this paper, we report that BPA exposure during the first days of embryonic life causes abnormalities of the inner ear, both in zebrafish, and in *Xenopus *embryos. The use of radioactive labeled bisphenols allowed us to determine the uptake of BPA and BPF and to estimate the capability of the embryos to biotransform and eliminate these xenobiotics. We found that the bio-concentration factors were 27 and 11 for BPA and BPF respectively. In addition we demonstrated that BPF but not BPA was metabolized in the zebrafish embryo. We used pharmacological approaches to test whether BPA action was linked to interferences with nuclear receptors such as ER and TR and found that BPA is not acting through these receptor systems during inner ear development. In contrast, we observed that the Na^+^/K^+ ^ATPase system is required for BPA action. Our results thus reveal an unanticipated effect of BPA during vertebrate embryonic development.

## Methods

### Fish stocks

Breeding zebrafish of the AB-TU and TU wild type strains were reared at 28.5°C and kept under a 8-hr dark/16-hr light cycle and staged as described [37]. All data were also obtained on the Konstanz wild type strain with identical results. The development of endogenous pigments was inhibited by exposing embryos to 1-phenyl-2-thiourea (PTU) at a final concentration of 0.2 mM. This PTU treatment was not included in experiments designed to assess the effects of different bispenols on pigmentation.

### Treatment of zebrafish embryos

Embryos (5 hpf) were exposed for 1 to 2 days to BPA (2,2-bis(4-hydroxyphenyl)propane, CAS No. 80-05-7), at concentrations ranging from 10^-8 ^M to 10^-4 ^M, 17 β-estradiol (E2) (from 1 nM to 0.1 μM), ICI 182.780 (ICI) (from 1 μM to 50 μM), TH (up to 10^-4 ^M), IOP (iodopropanoic acid, 5. 10^-3 ^M), PTU (5. 10^-3^) or ouabain (up to 2 mM) (Sigma) diluted in embryo medium, from a stock solution in ethanol, DMSO or distilled water. Wild type zebrafish treated by vehicule only were used as negative controls to compare effects on compound-treated zebrafish. Embryos (5 hpf) were also exposed for 1 to 2 days to 5.10^-5 ^M Bisphenol F (bis(4-hydroxyphenyl)methane), 7. 10^-5^M Bisphenol E (bis(4-hydroxyphenyl)ethane) and 10^-6 ^M Bisphenol C (1,1-Bis(4-hydroxyphenyl)-2,2-dichloroethylene) (Sigma), diluted in embryo medium, from a 10^-1 ^M stock solution in ethanol.

### Measurement of BPA and BPF uptake and metabolism by zebrafish embryos

[^14^C]-BPA, with a specific activity of 7.4 GBq.mmol^-1 ^and a radio-purity greater than 99% was purchased from Moravek Biochemicals (CA). [^3^H]-BPF, with a specific activity of 300 MBq.mmol^-1 ^and a radiopurity higher than 99% was purchased from Izotop (Budapest, Hungary). Zebrafish embryos (7 hpf, n=60 per group) were exposed for 72 h to 5.10^-5 ^M radiolabeled BPA, as described above. At 18, 36 and 72 h, water (1 mL) and embryos (3 pools of 4 individuals) were sampled separately for radioactivity measurement. Embryos were rinsed twice with E3 1X medium and weighed before analysis. At the end of the experiment, remaining water and embryos were collected separately and stored at -20°C until radio-chromatography analysis. The same experiment was repeated with radiolabeled BPF. The radioactivity present in embryos was measured by liquid scintillation counting with a Packard Tricarb 4430 counter after 90 min treatment at 60°C by 1 mL Soluene (Packard Instruments Co., Meriden, CT). Radioactivity in water was directly measured by liquid scintillation counting. For all vials, sample quenching was compensated by the use of quench curves and external standardisation. At the end of the experiment, remaining water and embryos were collected separately and stored at -20°C until radio-chromatography analysis. HPLC coupled to online radioactivity detection (HP 1100 coupled to Flo-One A500 detector, with Flo-scint II as scintillation cocktail, (Packard Instruments Co., Meriden, CT) was used for metabolite profiling. Water samples were evaporated to dryness and the residue was dissolved in the HPLC mobile phase before analysis. Radioactivity was extracted from pooled embryos with acetonitrile. The organic fraction was evaporated to dryness and redissolved in the HPLC mobile phase before analysis. HPLC conditions were as described by Zalko et al. [38] for BPA and by Cabaton et al. [39] for BPF. In order to control the stability of tested compounds in the medium, a blank, consisting of 50 mL embryo medium and 5.10^-5 ^M radiolabelled BPF or BPA was stored during 72 h at 28.5°C before radio-HPLC analysis.

### *Treatment of *Xenopus *embryos*

Groups of *Xenopus *embryos (n = 30 per group) at stage NF 18 [40] were placed in six well plates with 15 embryos per well (well volume 10 mL) and exposed for 48 hours to BPA (5 or 10 μM) or solvent (DMSO 1 μL in 10 mL). *Xenopus *embryos were left in contact with BPA for 48 h without any medium change and then washed and left to develop for another 48 h and being examined at stage 45. Embryos were fixed in 4% paraformaldehyde and photographed under either a Leica dissecting binocular microscope (upper panel) or Nikon SMZ-U microscope. (Lower panel).

### In situ hybridization immunohistochemistry and photography

Whole-mount *in situ *hybridization of zebrafish embryos was performed as described by [41]. Immunohistochemistry was performed as previously described [42] using the acetylated tubulin primary antibody (Sigma). Stained embryos were photographed with an AXIOIMAGER microscope (zeiss) or an Olympus BX51 microscope. Live embryos were photographed using Olympus SZX16 macroscope. Images were processed using the adobe photoshop software.

### Transient transfections

HeLa cells were maintained at 37°C in 5% CO_2 _in DMEM-F12 (GIBCO), 5% foetal calf serum (FCS). 24 hours before transfection, cells were plated in 96-well plates (20,000 per well) in DMEM-F12 (Invitrogen), 3% Dextran-Coated Charcoal Serum (DCC) whithout red phenol. Cells were transfected with Jet-PEI according with the manufacturer's instructions (Ozyme, Saint-Quentin-en-Yvelines Cedex France). Per well, 25 ng of the pSG5 expression vector for the ERα, ERβ-A and ERβ-B, 100 ng of the estrogen-dependentluciferase reporter construct (ERE-β-globin-Luc) and 50 ng of CMV-β-galactosidase plasmid (pCMV-bGal)were transfected. 48 hours after transfection, cells were harvested in a lysis buffer [25 mM Tris-phosphate (pH 7,8), 2 mM DTT, 2 mM EDTA, 10% glycerol and 1% Triton X-100] and the luciferase activity was measured with the luciferin solution [2 mM Tricine (pH 7.8), 1.07 mM (MgCO3) 4Mg(OH)_2_, 2.67 mM MgSO4, 0.2 mM EDTA, 0.53 mM ATP, 0.27 mM CoA and 0.48 mM luciferin] on a luminometer. pCMV-βGal was used to normalize the transfection efficiency.

### Limited proteolysis assay

Assays were performed as described in [43]. Proteins were synthesized *in vitro *using a TNT coupled Reticulocyte Lysate System kit (Promega) and labeled with [^35^S] methionine (Amersham Biosciences) according to the manufacturer's recommendations. BPA was incubated with the protein at different concentrations (from 10 nM to 1 μM). After incubation at room temperature for 15 minutes with ligand, receptor proteins were digested at room temperature for 10 minutes with trypsin. After this incubation time, buffer was added and incubated the mixture for 10 minutes at 100°C. SDS-PAGE was performed using a 12% gel. Electrophoresis was carried out using a BioRad gel apparatus at 170 V for 50 minutes and the polyacrylamide gel was visualized by autoradiography.

### In vivo *luciferase assay*

We used the transgenic ERE-Luc fish line described by Legler et al. [44]. Male fish with a weight of 300 mg (three to six months old) were exposed for 48 hours to one ligand or to a mixture of two ligands in glass aquaria (E2, ICI 182,780 and/or BPA). Ligands were dissolved in ethanol at concentrations of 10^-1 ^M or 10^-3 ^M and titrated to final concentrations not exceeding a solvent concentration of 0.01%. Single fish were anaesthetized (0.6 mM Tricain methanesulfonate) and transferred to an Eppendorf tube. Then, 135 mL of ice cold Triton-lysis buffer (1% triton-X-100; 15 mM MgSO_4_; 4 mM EGTA pH 7.0; 35 mM glycylglycine pH 7.8; 1 mM DTT) was added to each tube following homogenization using a micropestle. After centrifugation the supernatant was transferred to a new tube and extracts were measured in luminometric reporter gene assays carried out in duplicates in a Microplate Luminometer (Anthos). Light units from extracts of ligand-exposed fish and from non-exposed fish were used to calculate fold inductions.

## Results

### BPA exposure during early embryogenesis induces abnormalities of the otoliths

**Figure 1:**
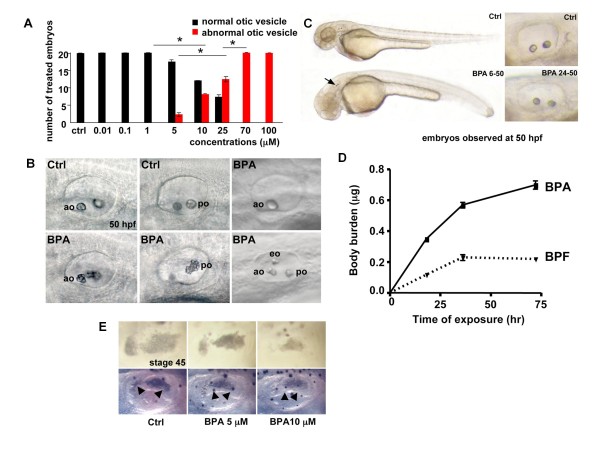
**BPA treatment induces otolith abnormalities**. **A**: BPA induced otolith malformation is dose dependent. Embryos were treated with different concentrations of BPA from 5 hpf onwards. Black bars represent embryos showing no otolith abnormalities, red bars represent treated embryos showing otolith abnormalities. (*: P < 0.01, Fisher test after Bonferroni correction). **B**: Anterior otolith (ao, upper left) and posterior otolith (po, upper middle) of control embryo and ao (lower left) and po (lower middle) of embryos treated with 70 μM BPA from 5 to 50 hpf showing otolith aggregation. In some rare cases a single otolith (upper right) or extra otolith (eo lower right) are observed. **C**: Upper panel: control embryo at 50 hpf (Upper right: close up of the otic vesicle). Lower panel: overall shape of BPA treated embryos form 6-50 hpf resembles control embryos, but display otolith aggregates (arrow). Lower right: close up of the otic vesicle in a BPA treated embryo form 24-50 hpf showing normal otoliths. **D**: After 75 hours of exposure, the concentration of BPA measured in embryos is 0.7 μg equivalent per mg fresh weight and 0.22 μg of BPF equivalent per mg fresh weight; see also Figure 2 C-E. **E**: BPA induces malformation of the otic vesicle in *Xenopus *embryos. *Xenopus *embryos were exposed to BPA (5 or 10 μM) from stage NF 18 to stage NF 40 (48 h) and examined at stage NF 45. The upper panel shows the morphology of the otoliths (or otoconia [73]) in control and BPA treated embryos. Note that 5 and 10 μM of BPA induce a progressive reduction in the size of the otoconia. The lower panel shows the semi-circular canals with a reduction in the distance between the two semi circular canals (arrowheads) induced by BPA. Similarly, the morphology of the developing semi circular canals is flattened under BPA treatment.

Zebrafish embryos were treated from 5 hours post-fertilization (hpf) onwards with concentrations of BPA ranging from 0.01 μM to 100 μM. While exposure to BPA <5 μM did not induced phenotypic abnormalities, treatment with higher doses induced otolith malformations (Figure 1A). These effects were seen in a small proportion (<5%) of embryos at 5 μM and increased at higher doses with more than 50% of affected embryos at 25 μM BPA. Notably, 100 μM BPA induced embryonic mortality probably linked to cardiac edema and cessation of blood flow. Abnormalities included aggregation of the otoliths, appearance of extra otoliths or their absence (Figure 1B). The vast majority of embryos exhibit bilateral aggregation of both anterior and posterior otoliths (< 18 otoliths forming an aggregate in the posterior otolith, Figure 1B). In some cases, altered semi-circular canals development was observed, but as this effect was highly variable it was not further characterized. In addition we observed that BPA had a mild effect on decreasing the general embryo pigmentation (not shown).

Otoliths (otoconia in higher vertebrates) are composed of calcium carbonate (CaCO_3_) and are required for normal balance and gravity sensing. Otolith formation requires bringing together organic and inorganic components in time and space. Disruption of any of these processes can lead to the formation of missing, ectopic or abnormally shaped otoliths [45]. Otolith defects in BPA treated embryos have not been reported in earlier studies monitoring BPA defects during vertebrate embryogenesis [32,33,46]. The BPA-induced phenotype is restricted to the otolith as the general shape of the embryos is not affected even if in high dose treatments we detect cardiac edema and stop of blood flow as previously observed [32] (Figure 1C). Moreover late BPA exposure, after 24 hpf, does not alter anymore the development of the otoliths (Figure 1C right panels).

In these types of studies, information on the bioavailability, biotransformation and excretion of the tested chemical are crucial to relate the waterborne exposure levels to the actual internal dose reaching the biological targets. Because limited data exist on the fate of bisphenols in fish embryos, we determined the uptake and metabolic profile of BPA and BPF (two bisphenols commercially available as radio-chemicals) in zebrafish embryos. To this end, 60 zebrafish embryos were incubated in a volume of 50 mL of E3 1X medium in presence of 50 μM [^14^C]-BPA or 50 μM [^3^H]-BPF for 72 hours. Embryos and medium samples were collected for radioactivity measurements and radio-HPLC analysis. As shown in Figure 1D, the concentrations of bisphenols measured in embryos at the end of the experiment corresponded to 0.7 μg BPA equivalent per mg fresh weight and 0.22 μg BPF equivalent per mg fresh weight, respectively. These values indicate that the concentration of radiolabeled compounds was 27 and 11 fold higher in embryos than in water, respectively. This result allowed us to estimate that the levels of BPA and BPF accumulating in the embryos were in the order of 10^-3^M and 5x10^-4^M, respectively.

Radio-HPLC profiles obtained from BPA exposed embryos and from the water, exhibited a single radioactive peak, displaying the same retention time as BPA itself (Additional file 1). In addition no metabolic products were found in the water, suggesting that at this late stage of development, zebrafish embryos were unable to biotransform BPA. In contrast, BPF was biotransformed by embryos, as shown by the radio-chromatograms obtained for embryos extracts and E3 medium samples, respectively (Additional file 1). Due to the low amount of available material, further investigations on BPF metabolite structures could not be carried out.

We next examined whether BPA exposure had an effect on otic vesicle development in another vertebrate embryo. The *Xenopus *embryo was selected because, like the zebrafish, effects on early development can be easily followed in this transparent, free-living embryo. *Xenopus *embryos were exposed to BPA during early development (stages 18-40) for 48 hours and examined at stage 45. Exposure to either 5 μM or 10 μM BPA caused a strong dose-dependent reduction in the development of the otoconia (*i.e. Xenopus *otoliths) (Figure 1D). We also observed a severe reduction of the semi-circular canals, with the presumptive arch of the canals being flattened by increasing doses of BPA (lower panel, Figure 1E).

### BPA effect is time and compound-specific

**Figure 2:**
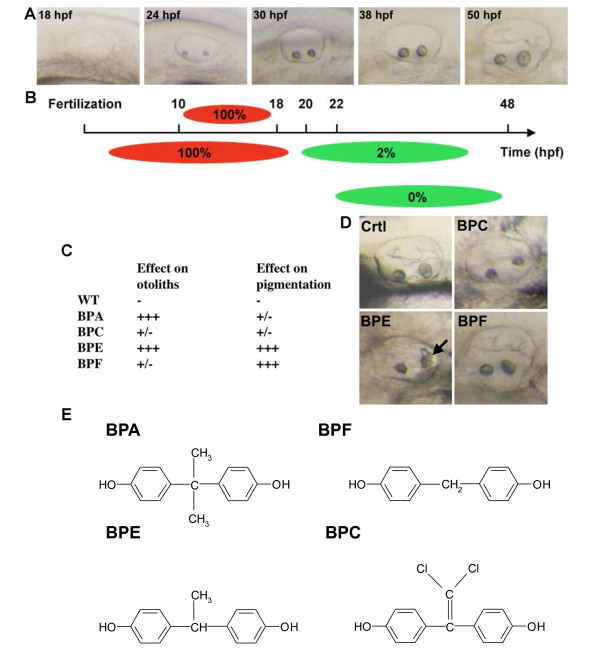
**Bisphenol effects are time and compound specific**. **A**: Live pictures of the developing otic vesicle from 18 to 50 hpf in zebrafish embryo. **B**: BPA affects otolith in a restricted time window. Diagram showing the effect of 70 μM BPA treatments with different starting points or length of exposure. Upper panel: BPA pulse treatments (red bars) were performed from 10 hpf. At different stage of development (18, 24, 30 and 38 hpf) embryos were washed and developed in BPA free medium (gray bars). Otic vesicles were scored at 50 hpf. Lower panel: BPA acts prior 22 hpf to induce otolith defects. Treatments started prior or at 18 hpf lead to 100% of otolith defects. Treatment started from 20 hpf onwards lead to 85% of embryos with otolith defect. Treatment started at 22 hpf lead to only 2% of affected embryos. Treatments started later did not lead to any otolith defect. Red bars represent time when embryos were exposed to BPA. Gray bars represent time when embryos were not exposed to this compound. **C**: Various bisphenols affect otolith development and/or pigmentation. Embryos were treated from 5 to 72 hpf and malformed otolith or pigmentation defects scored (+++ indicates maximum defect, i.e. malformed otolith or total lack of pigment). Treatment of embryos with either BPA (70 μM) or BPE (70 μM) resulted in malformed otoliths. BPE also affected pigmentation, as did BPF (50 μM). BPC (70 μM) was without clear effects in these assays. **D**: Pictures of the effect of BPC, BPE and BPF on otolith development. All embryos were exposed to 70 μM of bisphenol. Note that only BPE gives an otolith phenotype similar to what in observed in BPA treated embryos with otolith aggregates marked by a black arrow. **E**: Chemical structures of BPA, BPC, BPE and BPF.

To determine if the timing of BPA action corresponds to that of inner ear organogenesis in zebrafish, two series of experiments were done. First, BPA pulse treatments of different lengths were started at 10 hpf and continued until 18, 24, 30 or 38 hpf, a time frame covering the main steps of otic vesicle development that are shown on Figure 2A[47]. The morphology of the otoliths was examined at 50 hpf. In all cases, exposure to BPA (70 μM) during these critical periods induced otolith malformation with 100% of the treated embryos displaying otolith aggregates (Figure 2B, Upper red bars, n = 30). Otolith aggregates were never observed in control embryos (n = 30). In a second series of experiments, treatment started from 6 hpf onwards or at 2 h intervals thereafter till 30 hpf (ie earliest treatment starting at 6 hpf and latest treatment starting at 30 hpf). Again, the otolith morphology was examined at 50 hpf. When BPA exposure began prior to or at 18 hpf, 100% of the embryos displayed malformed otoliths (i.e. otolith aggregates). When treatment began at 20 hpf the proportion of affected embryos decreased to 85% (n = 40, Figure 2B, lower panel). However, almost no effects (only 2% of the embryos affected, n = 40) were seen when treatments began at 22 hpf (Figure 2B, lower panel). Treatments starting after 22 hpf did not induce any otolith defect (Figure 2B, lower panel and data not shown). Of note is the fact that phenotype severity (i.e; number of aggregates) is dependent on time of treatment onset. The earlier the BPA exposure (i.e. the closer to mid-blastula transition, 6 hpf), the stronger the phenotype (See Additional file 2). According to Riley and colleagues [48] and Colantonio et al., [49], otic vesicle formation occurs at 18-18.5 hpf. Thus, our results suggest that BPA acts prior to actual otolith formation per se, potentially acting on determination of cells that will give rise to the otoliths.

To test for the specificity of the observed phenotype, we tested the effects of other bisphenols. Zebrafish embryos were exposed to BPC, BPE or BPF. As BPA weakly reduces pigmentation, the effects of each bisphenol were recorded on otolith development and pigmentation (Figure 2C). We observed differential effects of each bisphenol on these endpoints with BPE producing the most marked effects on both processes (Figure 2C, D arrow). BPC was least disruptive, affecting neither otolith nor pigmentation. Interestingly, we observed that the presence of methyl groups on the central carbon between the two phenols had no effect on the action of the molecule on the otic vesicle (e.g. BPA, BPE and BPF are active). In contrast, the presence of a dichloroethylene group on the central carbon (e.g. BPC) abrogates the disrupting effects of bisphenol (Figure 2E).

Taken together these results show that although the phenotypic effects are observed only at high concentrations, they disrupt a precise developmental pathway. Indeed, the time dependency and the chemical specificity suggest that this is not a general toxicity effect.

### Developmental analysis of the BPA-induced otolith phenotype

In order to gain insight into the developmental origin of the phenotype, we compared the expression of several markers of otic vesicle and otolith formation between control and BPA-treated fish. First, we monitored if the phenotype of BPA treatment was linked to a developmental delay using a marker, *pax2.a*, that shows a normal pattern (revealing only a smaller otic vesicle at 18 hpf, data not shown).

**Figure 3:**
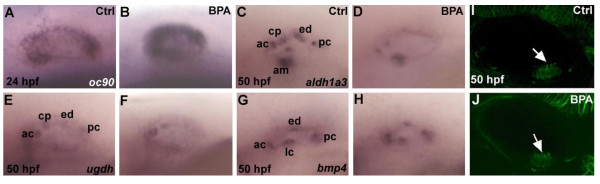
**Expression of markers of inner ear development in zebrafish embryos are affected by BPA treatment**. **(A,B)***oc90 *a gene require for otolith formation in zebrafish is up-regulated under BPA treatment at 24 hpf (n=30). In situ for control and BPA treated embryos were performed in the same tube with the tip of the tail removed for the control embryos. **(C,D)***aldh1a3 *is detected in the anterior cristae (ac), the cranial epithelial projection (cp), the endolymphatic duct (ed), the posterior cristae (pc) and the anterior macula (am) in wild type zebrafish embryo at 50 hpf (M), its expression is reduced in ac, cp and am and is absent in ed and pc in BPA treated embryos from 6 hpf onward **(D)**. **(E,F)** Expression of *ugdh *at 50 hpf in control otic vesicle **(E)** is detected in the ac, cp, ed and pc. In BPA treated embryos from 6 hpf onwards **(D)**, *ugdh *expression is severely reduced and remains solely detected in the ac and the cp. **(G,H)** Expression of *bmp4 *in control embryos at at 50 hpf **(G)** in the anterior (ac) lateral (lc) in the posterior cristae (pc) and the endolymphatic duct (ed) remains unaffected after BPA treatment **(H)**. **(I,J)** Confocal microscopy of acetulated tubulin antibody staining showing the presence of the ciliated macula (white arrow) in control embryos** (I)** and in BPA treated embryos (white arrow in **J**).

Next, we checked whether the phenotype implicated a defect in hindbrain patterning as this can occur when specific pathways (e.g. FGF, RA) are disrupted [50,51]. The expression of *egr2a*, a marker of rhombomere 3 and 5 segmentation at 18 hpf was thus examined. *egr2a *expression is not affected (Additional file 3A,B), suggesting that hindbrain patterning in the region of the otic vesicle is unaltered by the BPA treatment. We next tested if the antero-posterior or the dorso-ventral patterning of the otic vesicle was affected by BPA treatment. Using *atonal1 *(*atoh1a*) and *hmx3*, two markers expressed in the anterior part of the otic vesicle at 26 hpf, we showed that the antero-posterior patterning is not affected in BPA treated embryos (Additional file 3C-F). Similarly, dorso-ventral patterning is not altered, as displayed by *dlx3b *labelling at 26 hpf (Additional file 3G and 3H) [52]. Recently, Petko and colleagues [53] identified *otoconin 90 (oc90) *as essential for normal otolith development during zebrafish embryogenesis. When translation of this gene was abolished, otoliths were smaller in size, altered in shape or absent. To test for possible links between the BPA-induced phenotype and the *oc90 *phenotype, we studied *oc90 *expression in BPA treated embryos at 24 hpf. In our hands, *oc90 *is expressed in the ventral portion of the otic epithelium (Figure 3A). In BPA-treated embryos a stronger signal of *oc90 *expression is detected (Figure 3B). A similar up -regulation of *oc90 *is observed in embryos treated with another bis-phenol leading to otolith malformation, BPE (Additional file 4). Therefore, we conclude that BPA acts by altering directly or indirectly the expression of genes implicated in otolith development. To better understand the relationship between *oc90 *and BPA action, we injected a published morpholino against the *oc90 *gene [53]. The most dominant phenotype observed is a single posterior otolith (Additional file 5C) with sometimes the presence of 2 or 3 small otoliths in contact with each other. This finding recalls those of Petko and colleagues [53]. However, when we treated these *oc90 *morphants with BPA we observed a full BPA phenotype (i.e. high numbers of otholith aggregates) in the posterior developing otolith (Figure 5D). Therefore, even in the absence of oc90, BPA exposure can lead to otolith aggregation in a similar fashion to control treated embryos (see Additional file 5B and 5D).

We then studied if and how the inner structures of the ear were affected by BPA treatment at later embryonic stages (50 hpf). To this end we analyzed the expression of *ugdh *[54,55], *aldh1a3 *[56] and *bmp4 *[56]. *ugdh *is expressed in specific regions of the otic vesicle (anterior and posterior cristae and endolymphatic duct and cranial epithelial projection [54]. In BPA treated embryos *ugdh *expression is severely reduced especially in cranial epithelial projections, endolymphatic duct and in the posterior cristae (Figure 3E and F) while expression persists in the anterior cristae. Similarly, *aldh1a3 *expression is decreased in endolymphatic duct and posterior cristae while still expressed in anterior cristae, the cranial epithelial projection and the anterior macula (Figure 3C and 3D). In contrast, *bmp4 *expression in the anterior lateral and posterior cristae is unaffected (Figure 3G-H). The otic vesicle is highly ciliated and the cilia are important for otolith formation. We thus decided to look at these cilia in BPA treated embryos, but did not detect any difference in number, shape nor size of the cilia of the macula in control embryos and BPA treated embryos (Figure 3I,J). This result correlates well with our *in situ *results as all markers of the anterior macula are expressed normally in BPA treated embryos (Figure 3D,H).

Taken together these data reveal that BPA has a broad effect on the specification of several inner ear territories. The morphological otolith phenotype is therefore only one aspect of the more general action of BPA in this organ (see Discussion).

### BPA effects on otolith development are estrogen-independent

BPA is known to act as a partial agonist of estrogen (E2) receptors (ERs) in mammalian systems [20-24]. In addition several recent observations suggest that both in mammals and fish estrogen signalling plays a role in hearing suggesting a possible connection with otic vesicle formation [57,58]. We questioned whether BPA is able to bind to the three zebrafish ERs (*esr*) and regulate transcription in transient transfection assays. A limited proteolysis assay was carried out using the three previously cloned receptors, *esr1*, *esr2a *and *esr2b *[59,60] of which *esr1 *and *esr2a *are ubiquitously expressed during zebrafish embryogenesis including in the otic vesicle [61]. As seen in Additional file 6, each of the three ERs is protected against trypsin degradation in a dose-dependent manner with both E2 and BPA. However, the affinity for E2 is three orders of magnitude greater with protection occurring at all E2 concentrations above 0.01 μM, whereas 1 μM to 10 μM BPA is required to protect from trypsin digestions. We thus conclude that BPA can bind all three ERs, albeit at low affinity.

The next question was whether ERs could be activated by BPA. Transient transfections were done using HeLa cells, which do not express endogenous ERs. These cells were co-transfected with a luciferase reporter gene under the control of consensus estrogen response element (ERE) along with an expression vector encoding one or the other of the ERs: Erα, Erβ-A and Erβ-B. Cells were exposed to either increasing concentrations of E2 or BPA (Additional file 6). We found that luciferase expression was induced in the presence of E2 and BPA, but that the ED50 for E2 was around 1 nM or 0.01 μM dependent on which of the ER was used, whereas the ED50 for BPA on each ER was 100 to 1000 times higher (1 μM) This result corroborates the results of the limited proteolysis assay described previously. Thus BPA binds and activates the three zebrafish ERs in the micromolar range.

To verify that BPA elicits a transcriptional response *in vivo *we used a transgenic reporter zebrafish line that contains a luciferase gene under the control of an ERE linked to a minimal promoter (3xERE-TATA-Luc) [44]. We performed this assay in juvenile fish (3 months old) since, in our hands, E2 did not induce a detectable luciferase response using these transgenic fish during embryonic stages [62]. The transgenic ERE-Luc fish were treated with BPA at 10 μM. The Luc activity was then measured from pooled pairs of juveniles. In presence of 1 μM E2, we observed a 620 fold induction of Luc activity, whereas, as expected, ICI 182,780 (ICI), does not induce any activation and completely abolishes E2 induced activity. In presence of 10 μM BPA, Luc activity is weakly induced (<7 fold), but no induction is detected when co-treated with 1 μM of ICI, clearly suggesting that this effect is ER-dependent. These results show that BPA can weakly activate the estrogen pathway *in vivo*.

**Figure 4:**
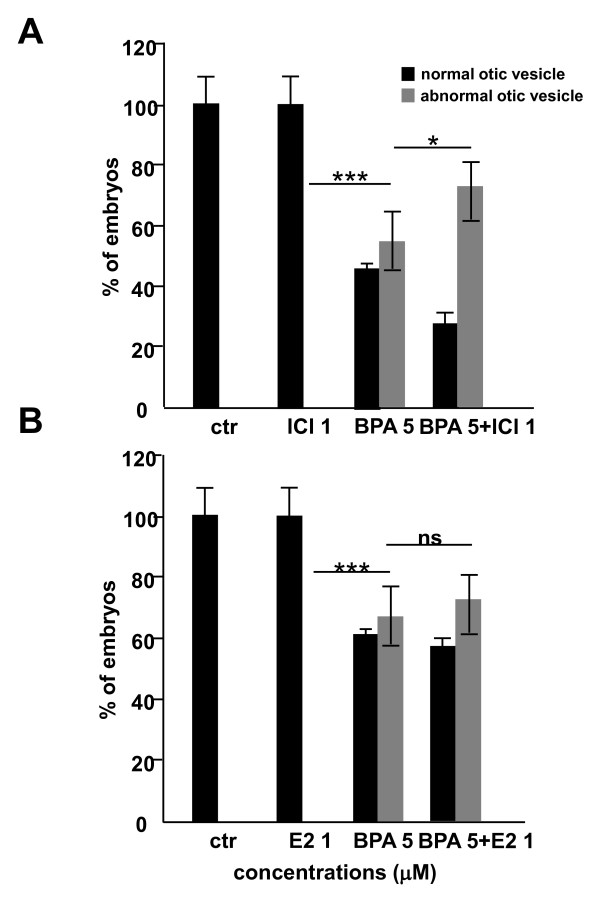
**BPA effect on otolith development is estrogen receptor-independent**. Morphology of the inner ear of zebrafish embryos at 48 hpf following exposure to BPA with or without ER antagonists (ICI 182 780) or agonists (17-β estradiol, E2). Treatment with either 1 μM ICI **(A) **or 1 μM 17-β estradiol **(B)** from 5 to 48 hpf does not induce any malformation of the developing semi-circular canals nor otoliths. Moreover, the BPA-induced otolith phenotype is not rescued when embryos are co-treated with BPA 5 μM +ICI 1 μM (A) or BPA 5 μM +βE2 1 μM **(B)**. Interestingly, co-treatment with BPA 5 μM +ICI 1 μM lead to in increased ratio of affected otolith than a treatment with BPA 5 μM alone **(A)**. On the other hand co-treatment with BPA 5 μM +βE2 1 μM gives a similar ratio than a treatment with BPA 5 μM alone **(B)**.

We next examined if the otolith phenotype was linked to an alteration of the estrogen signalling pathway. A first approach was to test whether the ER agonist, E2, or the ER antagonist, ICI, modified BPA effects on otolith morphology (Figure 4). Zebrafish embryos were exposed to BPA with or without either 1 μM E2 or 1 μM ICI. As shown in Figure 4A and B, neither ICI nor E2 treatment alone induced phenotypic alteration of the otoliths (0%, n=120). However, whereas E2 had absolutely no effect on BPA-induced phenotype in terms of morphology or frequency, ICI exhibited a certain level of synergy with BPA, since there were consistently more affected embryos after co-treatment than with BPA alone (Figure 4A; 56.7%, n = 120 of otoliths affected upon BPA exposure 5.10^-6 ^M alone versus 73.3%, n = 120 of otoliths affected upon BPA exposure 5.10^-6 ^M supplemented with ICI 10^-6 ^M). However, in this case the severity of the morphological phenotype was unaltered. Taken together, these results indicate that the BPA action on otic vesicle development is not mediated primarily by ERs but that, to some extent, modulation of ERs may alter the frequency of the BPA-induced effect.

### The BPA-induced otolith phenotype is TR-independent

BPA has been shown to be a TR antagonist in mammalian cells [28,30] and TR knock-out mice have a hearing defect linked to abnormalities of ear development [63,64]. More recently *in vivo *work demonstrated that BPA inhibits T3 signalling in *Xenopus *embryos [31] and prevents metamorphosis in *Xenopus *tadpoles [35]. As for the ZfERs, we tested if ZfTRs could be activated or inhibited by BPA. These data show that BPA was inactive on ZfTRaA being unable to induce the activation of the receptor or to inhibit its activity in presence of low amount of T3 (data not shown).

**Figure 5:**
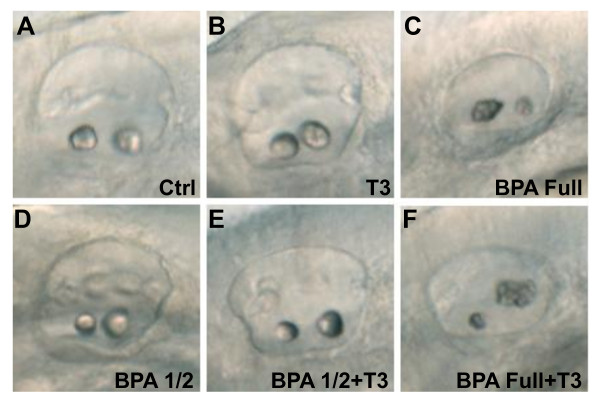
**BPA effect on otolith development is thyroid hormones independent**. **(A)** Control otic vesicle at 50 hpf with two otoliths. **(B)** Treatment with 1 μM of T3, from 5 hpf onwards lead to a normal otolith development. **(C)** 70 μM BPA treated embryo from 5 hpf onwards lead to otolith aggregates. **(D)** Half dose of BPA, 35 μM, does not affect otolith development. **(E)** Co-treatment of half dose of BPA, 35 μM, supplemented with 1 μM of T3 does not affect otolith development. **(F)** Co-treatment of full dose of BPA, 70 μM supplemented with 1 μM of T3 lead to a similar otolith phenotype than BPA 70 μM alone **(compare C with F)**.

To test the effect of BPA on the TH signaling pathway, we used a pharmacological approach to assess whether BPA action on zebrafish otolith implicated TH signalling. We first observed that treatment with high doses of T3 (1 μM) did not induce any obvious morphological abnormalities of the otoliths (Figure 5B). In order to prevent the rapid degradation of exogenous T3 by endogenous deiodinases, the experiment was repeated in the presence of two compounds that inhibit deiodinase activity, either iodopropanoic acid (IOP) or PTU [65]. Similar results were obtained with or without these agents. Treatment of 40 embryos with BPA 70 μM alone or BPA + T3 gave similar phenotypes (compare Figure 5C and 5F) and frequencies (data not shown). To carry out a more sensitive assessment of potential interactions between the two pathways, embryos were treated with suboptimal doses of BPA (35 μM, i.e. half the previous dose) and monitored to see if 1 μM T3 acted synergistically with BPA on the otolith phenotype. As found previously, co-treatment with T3 had no effect (compare Figure 5D and 5E). These data suggest that there are no interactions, neither positive nor negative between BPA target(s) and TH signalling.

### BPA and ouabain-induced otolith formation

**Figure 6:**
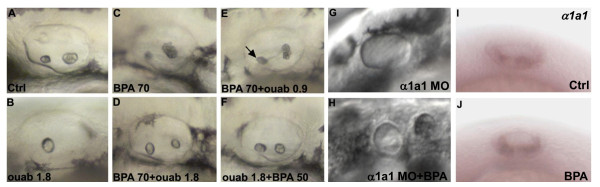
**Chemical inhibition of Na/K ATPase rescues the BPA induced phenotype**. **(A)** 50 hpf control embryo with two normal otolith. **(B)** Treatment with 1.8 mM ouabain from 6 hpf onwards leads to one misformed otolith. **(C)** Embryo treated with 70 μM BPA from 5 hpf onwards leads to otolith aggregates. **(D)** Co treatment with 70 μM BPA and 1.8 mM oubain from 6 hpf onwards leads to normal otolith. **(E)** Co treatment with 70 μM BPA and 0.9 mM oubain from 6 hpf onwards leads to a mild BPA like phenotype. In some cases one almost normal otolith is found (arrow). **(F)** Co treatment with 50 μM BPA and 1.8 mM oubain from 6 hpf onwards leads to two normal otoliths. **(G)***α1a1 *morphants display a complete absence of otolith. **(H)** 70 μM BPA of *α1a1 *morphants did not rescue otolith formation and result in a complete absence of otolith. **(I)***α1a1 *is expressed in the lower membrane of the otic vesicle at 24 hpf. **(J)** A similar expression of *α1a1 *is detected in BPA treated embryos.

One of the main enzymes involved in ion exchange and various aspects of inner ear formation are the ouabain sensitive Na+/K+ ATPases [54,66]. Addition of ouabain to embryos was used to examine whether the effects of BPA implicated Na+/K+ ATPases. Ouabain exposure (1.8 mM from 6 hpf onwards) caused aberrant development of otoliths, with either complete absence (20% of the embryos) or one misformed otolith (80%; see Figure 6B). Higher doses led to embryonic mortality at 48 hpf as reported by Blasiole et al. [54]. This phenotype could be viewed as opposite to that caused by BPA (at 70 μM from 6 hpf onwards) i.e. formation of multiple small aggregated otoliths (Figure 6C). Interestingly when we co-treated embryos with 1.8 mM ouabain + 70 μM BPA a normal otolith formation was observed (Figure 6D): two otoliths are present in these fish. We interpret this as a rescue of the BPA effect by ouabain treatment. This suggests that the two compounds have opposite effects: on the one hand, BPA promotes multiplication and aggregation of otoliths, whereas, inversely, ouabain blocks their formation. We thus explored the dose-dependency of this effect. Using a lower dose of ouabain (0.9 mM, half dose) induced only very limited rescue of the full dose BPA phenotype. However, with this half dose ouabain and full dose BPA embryos were less severely affected than the full dose BPA alone (Compare Figures 6C and 6E). In contrast, full dose ouabain plus lower dose BPA (50 μM) treated embryos also displayed normal otoliths, with two distinct otoliths while only one misshapen otolith resulted from full dose ouabain alone (Compare Figures 6F and 6B).

It has been recently reported that the morpholino knock-down of one subunit of Na+/K+ ATPase α 1a.1 (α *1a.1*) phenocopies the ouabain inhibition of otolith formation [54]. We thus tested if α *1a.1 *was required for the occurrence of the BPA phenotype. We observed the same phenotype as Blasiole et al. [54] after α *1a.1 *morpholino injection, i.e. a complete loss of otolith (Figure 6G). Treatment with 70 μM BPA of α *1a.1 *morphants (n = 20) gives rise to the same phenotype as α *1a.1 *morphants alone: the complete loss of otolith (Figure 6H). When a lower dose of morpholino is used, a single small otolith is observed (Additional file 7C). When morpholino treated embryos are treated with 70 μM of BPA, extra otoliths (2-3 only) are observed (Additional file 7D). However high level of otolith aggregates are never observed (compare Additional file 7B,D). This result suggests that a lower level of Na+/K+ ATPase alpha 1 can reduce the BPA-induced effect on the otolith, though this relationship needs further analysis (see Discussion).

One possibility to explain these results would be that BPA induces expression of *α1a.1*. We therefore tested if BPA treatment modulates *α1a.1 *expression at 24 hpf (Figure 6I and 6J). We found that it is not the case: *α1a.1 *is detected both in terms of localization and intensity in a similar way in control and in BPA treated embryos. Blasiole et al., [66] showed that three other Na+/K+ ATPases are expressed in the developing ear. We carried out *in situ *hybridization with probes for these three other Na+/K+ ATPase enzymes in BPA treated embryos. No effect on their expression was found compared to control embryos (data not shown). These data allow us to exclude the possibility that Na+/K+ ATPase genes are regulated at the transcriptional level by BPA. However, it remains possible that BPA interferes with Na+/K+ ATPase function in other indirect ways, e.g. by inhibiting its enzymatic activity, by interfering with the stability of the protein and/or its post-translational modifications or affecting sub-cellular distribution.

## Discussion

### BPA affects otolith development

Despite the fact that the effects of BPA on development have been the subject of numerous investigations, few experiments have explored effects during embryogenesis [18,19,36]. Here, the distinct advantages of the aquatic, free-living embryos used in this study come to the fore, notably their external and maternally-independent development, and their transparency that together facilitate investigation of morphological phenotypes. Indeed, the main result from our study is that BPA specifically affects otolith development during zebrafish and *Xenopus *development. The otolith phenotype that we observed is not linked to the cardiac effect previously observed since the timing of the two events differ, the otolith phenotype being dependent on treatments carried out during earlier developmental periods. Our data show that BPA acts early in development, during zebrafish embryogenesis, before 20 hpf to affect otolith formation and that this effect is specific to BPA and closely related bisphenols, since another molecule of the same class (e.g. BPC) does not have the same effect. This observation underscores the possible relevance of the phenotype we describe.

It is interesting to note that the timing of BPA exposure resulting in otolith phenotype actually corresponded to the timing of otic vesicle organogenesis. The otic placode becomes visible at approximately 16 hpf and forms a vesicle with a lumen by cavitation at approximately 18 hpf [67] (see Figure 2A). Two otoliths appear in the lumen by 19.5 hpf, and at about 24 hpf, the first sensory hair cells are seen, grouped in two small patches, one beneath each otolith, corresponding to the future macula. We found that BPA affects inner ear development in a precise window, at the time of the first sign of morphological appearance of the otoliths, that is at 20 hpf. Indeed, if BPA treatment was delayed till just 22 or 24 hpf, no effects on otolith formation were found. This precise timing of BPA action is indicative of an interaction with specific targets active during early otolith development. These observations emphasize that the effects are not due to acute toxicity but are due to a specific interference with a developmental process. The fact that other bisphenols are inactive also suggests that BPA interacts with a precise selectivity on specific targets. In addition no sign of acute cellular stress such as apoptotic cells or necrotic patches were detected in the embryos after BPA exposure (data not shown).

It is important to note that BPA induces abnormalities of otolith development in two distant vertebrate species, zebrafish and *Xenopus*. Interestingly, the two phenotypes are different. In zebrafish we observed aggregates of otoliths with sometimes loss of one of the otolith. In contrast, in *Xenopus *BPA induces a strong dose-dependent reduction in the development of otoliths and a severe reduction of the semi-circular canals. In zebrafish we sometimes observed abnormalities of semicircular canals but with variable penetrance. We consider that this late phenotype may be a consequence of an early defect. In *Xenopus*, BPA was recently shown to induce malformations of the head region [36]. In this study, treatment with 25 or 50 μM of BPA resulted in scoliosis and malformation of the head region including a shortened distance between the eyes. Here, we observed a similar phenotype when BPA treatment was begun at the egg stage at these concentrations.

Our experiments reveal that only some bisphenols affect otolith development. BPA, BPE and to a lesser extent BPF induced otolith phenotypes whereas BPC was inactive. These differences could be attributed at least in part to dissimilarities in terms of bioavailability and metabolism of bisphenols in embryos, resulting in variations in concentrations of these bisphenols in target tissues. Comparing the fate of BPA and BPF, the bio-concentration and biotransformation of these compounds differed substantially. Residual levels of BPA in embryos were 2.5 fold higher than calculated for BPF. Furthermore, in the case of BPA no break-down products were found, as most of BPF was fully metabolized by the end of the experiment. Due to methodological limitations, these metabolic analyses could not be extended to other bisphenols. However, the data indicate that the extent of the phenotypic effects may be related to the uptake, biotransformation and elimination of these chemicals by zebrafish embryos. Perez et al. [14], using MCF7 human breast cancer cells in culture, demonstrated that the estrogenicity of bisphenols was influenced not only by the length of the substituents at the bridging carbon, but also by their nature. In a yeast two-hybrid assay, Chen et al. [13] ranked the estrogenicity of bisphenols as follows: BPB > BPP > BPA > BPE > BPF > BPS. Interestingly we found that some of these compounds also act to reduce pigmentation and that the pharmacology of this effect is clearly different: for pigmentation BPE and BPF are clearly the most active compounds, followed by BPC and BPA that show modest effects. These data suggest that bisphenols interfere with different targets in the otoliths and in the pigmentation developmental pathways.

The lowest concentration at which an effect was observed was 5 μM, but with low penetrance (less than 5% affected embryos). At 25 μM the majority of the embryos were affected and all at 70 μM. The lowest active concentration (5 μM) corresponds to more than 1 mg/mL several orders of magnitude higher than the levels reported in aquatic environments [4-6] or in human serum [8,9]. Of note however is the fact that the concentration of BPA found in amniotic fluid was approximately five-fold higher than levels measured in maternal plasma [10]. Even these high concentrations however are markedly lower than the one needed in our assay to generate a phenotype. Thus, the effect we observe in the aquatic species is probably not relevant to human populations.

### A previously undescribed otolith aggregate phenotype

The development of the inner ear in fish and amphibians is representative of the development of inner ears of vertebrates in general. Vertebrate inner ear development is a self-contained model system for fundamental research into genetic control of development and, as shown here, for detecting the effects of potential EDCs on early vertebrate development. Whitfield et al. [47] carried out a large scale screen of zebrafish mutants induced by N-ethyl-N-nitrosurea treatment. Mutations occurring in no less than 58 genes leading to defects in the development of either the semicircular canals and/or the otoliths were identified [47]. In this screen the authors did not examine fine morphology of the inner ear, such as hair cells, and so they estimate that many more genes are potentially involved in the process.

Interestingly, BPA induces a new zebrafish otolith phenotype. Most of the phenotypes of otolith formation are caused by the specific knock-down or mutation of a given gene [47,68]. Many of them result in a small malformed ear, a defect in the AP or DV axis, or in specific deletion of specific structures (semicircular canals, otoliths) but to our knowledge the phenotype we describe here has not been previously described. The abnormalities we observe included aggregation of up to 18 otoliths (Figure 1B, lower panel), appearance of multiple otoliths or their absence (Figure 1B). It is clear that the majority of the embryos exhibit a bilateral aggregation of both the anterior and posterior otoliths. This is clearly different (or opposite to) to the loss of otolith observed by Blasiole et al. [54] after treatment with ouabain or morpholino knock-down of the Na+/K+ ATPase α1a.1. In fact the phenotype that we find most similar to the effect of BPA is the one seen after inhibition of the *oc90 *gene, a member of PLA2-like otoconin family [53]. It is interesting to note that *oc90 *is clearly upregulated in the otic vesicle after BPA treatment suggesting it may be play an important role in generating the BPA phenotype. Nevertheless we still observed the otolith aggregation induced by BPA following *oc90 *knock-down, suggesting that this gene is not required for BPA action. Given the fact that BPA up-regulates *oc90 *expression we cannot exclude that *oc90 *act as a modifier of BPA action but this remains to be addressed experimentally.

The developmental basis of the phenotype was analyzed by studying the expression of gene markers. We found that the patterning of the otic vesicle placode and the hindbrain segmentation are normal and that the antero-posterior and dorso-ventral axes of the otic vesicle are correctly patterned. Thus, despite the fact that BPA acts prior to 20 hpf we only detect defects with gene markers at later stages, suggesting these modified expressions derive from early action of BPA on an unknown target. The change of gene markers observed suggests that the phenotype extends much further than a simple abnormality in otolith formation. Indeed, with *aldh1a3 *and/or *ugdh *we observed a loss of expression in specific regions such as cranial epithelial projections, endolymphatic duct and in the posterior cristae. This result is in accordance with the fact that we often see, with a variable penetrance, morphological alterations of the semicircular canals. Of note are the changes of expression observed in otic vesicle markers after BPA treatment, which are reminiscent of the changes reported by Petko et al., [53] after *oc90 *inhibition, with a normal *msxC *and *dlx3b *expression in both cases. The early timing of the effects in our data suggest that BPA exposure can have multiple consequences for the late development of the ear, resulting in abnormal formation of the otolith as well as other defects such as in the semicircular canals. Unfortunately, results from the *in situ *analysis do not really clarify the causes of the dramatic phenotype in otolith aggregates. Up-regulation of *oc90 *might be a determinant factor for this phenotype. In fish, otoliths are composed of calcium carbonate crystals condensed on a core protein matrix. *oc90*, is the ortholog of the mammalian *otoconin-90 *gene which encodes the major matrix protein of otoconia. Therefore it is tempting to speculate the with an excess of signal that will lead to the development of the otolith the consequences will be an excess of otolith as observed in our BPA treated embryos. The precise mechanisms underlying this effect have yet to be understood.

### Interaction with nuclear receptor pathways

Much data in the literature show that BPA interferes with the estrogen signalling pathway through direct binding to ERα or ERβ and thereby induce an ER agonist or SERM (Selective Estrogen Receptor Modulator) [19]. We have indeed verified that BPA binds to and positively regulates the activity of the three ERs present in zebrafish, although at higher concentration (ED_50 _at the micromolar range) than in mammals [22].

Estrogen receptor signalling is intricately implicated in the development, function and regeneration of the inner ear [69]. It was therefore a reasonable working hypothesis that the effects of BPA implicated interference with ER signalling during otic vesicle development. We tested this hypothesis in three ways. First, we examined the effects of estrogen agonist and antagonist activity on otolith development either alone or in combination with effective doses of BPA. We observed only a weak increase of the cotreatment of BPA and ICI on the number of affected fish (see below). Secondly, we studied if BPA in zebrafish could activate an ERE-Luc transgenic reporter gene [44]. We found a modest effect at the concentrations that clearly induce the phenotype (10 μM). These data thus suggest that BPA does not act in an estrogen receptor-dependent manner during otolith development. Several recent observations in the literature indeed suggest that BPA have a much broader set of targets than previously expected. For example, recently the genes responding in male zebrafish liver to the exposure to 17 β-estradiol, and BPA were identified through a microarray analysis [70]. Interestingly these data reveal that the transcriptional network regulated by 17 β-estradiol and BPA in zebrafish are very different. These observations are fully consistent with estrogen receptor-independent effects of BPA that we describe here through developmental analysis. The fact that we observed a synergy when we treated the fish with BPA and with the anti-estrogen ICI suggests a possible connection of the BPA targets and estrogen signalling. This idea is consistent with several recent observations suggest that both in mammals and fish the estrogen signalling play a role in hearing, suggesting a possible connection with otic vesicle formation [57,58]. The molecular and developmental basis of this cross-talk remains to be explored.

Several data have recently suggested that both TR isoforms (TRα and TRβ) can be targets of BPA (reviewed in [28]). In transient transfection experiments, BPA (in the micromolar range) suppressed T3-stimulated transcriptional activity stimulated in a dose dependent manner. Since T3 and TRβ are important for ear development [63,64] we thus tested if the action of BPA in zebrafish otolith development could be linked to its interference with the TR signalling system. We observed no obvious interaction between BPA, T3 and otolith formation: the addition of 1 μM T3 did not interfere, either positively or negatively, with the phenotype induced by BPA. BPA was tested at an optimal (70 μM) or a suboptimal (35 μM) dose of BPA and in none of these situations did we observe an effect of added T3. Thus, interference with the TH signaling pathway does not seem to be responsible for the BPA-induced phenotype. However, induction of deactivating deiodinases by the exogenous T3 cannot be excluded, a phenomenon that would neutralize the exogenous T3. The lack of BPA interaction with TH signalling is in accordance with our previous observation that altered expression patterns of TRs during otic vesicle development in zebrafish could not be detected [61].

### The BPA-induced phenotype and Na^+^/K^+ ^ATPase activity

Na^+^/K^+ ^ATPase are key enzymes implicated in cell homeostasis by the maintenance of electrochemical gradients. Na^+^/K^+ ^ATPase activity has been implicated in otic vesicle formation and otolith development shown by treatment with a chemical inhibitor and morpholinos that inhibit a subunit of this enzyme [54,66]. The Na^+^/K^+ ^ATPase enzyme is composed of functionally distinct subunits each with multiple isoforms encoded by different genes [54]. In the case of inner ear development, inhibition of Na^+^/K^+ ^ATPase subunits interferes with otolith and semicircular canal biogenesis, though the mechanisms implicated are still unclear [54]. Numerous cell types are implicated in the formation of otoliths [71]. In fish, otoliths are secreted into the otocyst (the part of the inner ear containing the otoliths) then captured by cilia of specialised cells that tether the anterior and posterior otoliths in place at each pole of the otocyst. The first crystals tethered serve as seeds for further growth. Na^+^/K^+ ^ATPase could be required for determining electrochemical gradients during various parts of these processes. In the growth of the semicircular canals, Na^+^/K^+ ^ATPase subunits are expressed in the areas that give rise to the protrusions that determine their growth.

In our experiments both BPA and ouabain, the inhibitor of Na^+^/K^+ ^ATPase, induced opposite phenotypes and combination of lower doses of each chemical rescued otolith formation. This result could indicate that the effects of BPA are dependent on an Na^+^/K^+ ^ATPase activity and one of the heterodimeric enzymes expressed in ear. To test this hypothesis knock-down of *α1a.1*, the major Na^+^/K^+ ^ATPase subunit expressed in the otic vesicle was carried out and showed that, indeed, the BPA phenotype cannot be observed without this enzyme. Nevertheless this effect is difficult to interpret since it may be simply be linked to the inability of *α1a.1*-morphants embryo to form otoliths properly. Combined with the pharmacological interaction noted above (BPA rescues the effect of ouabain; Figure 6), a possible scenario is that *α1a.1*, and more generally Na^+^/K^+ ^ATPase activity act downstream of BPA target genes in the otic vesicle. However, this is not the case as we saw no effects of BPA exposure on the expression of *α1a.1 *or on other Na^+^/K^+ ^ATPase subunits (Figure 6 and data not shown). It is also possible that the two pathways are independent. If *α1a.1 *is strictly required for otolith formation, the morphogenesis defects induced by BPA cannot be observed in the absence of otolith. By injecting a sub-optimal dose of *α1a.1 *the signalling cascade dependent on *α1a.1 *can be activated causing a small otolith to form. Once formation is initiated, this otolith treated with BPA will still form an aggregate but due do a low level of α1a.1protein present in the embryo, due to morpholino injection, the aggregate phenotype is mild compared to wild type. This scenario implies that the effect of BPA on Na^+^/K^+ ^ATPase and otolith formation is indirect. However, if this were the case one would not see a rescuing effect of BPA and ouabain combination that we observed. Taken together, these results suggest a link between BPA and Na^+^/K^+ ^ATPase during induction of otolith malformation, but the precise molecular nature of this link and the exact degree of this dependency remains to be explored.

### Conclusion

In this manuscript we show that BPA affects otolith development in two vertebrate species, zebrafish and *Xenopus*. In both cases the deleterious effects are limited to a time window corresponding to the period of otic vesicle development. Moreover, neither estrogen nor estrogen antagonists, nor TH modify the BPA response, underscoring the concept that the effects observed are estrogen and TH-independent. We conclude that vertebrate organogenesis can be modified by BPA through novel mechanisms. A clear link with the Na^+^/K^+ ^ATPase system was observed. Our experiments provide an example of a developmentally relevant defect generated by BPA treatment that is independent of its main mode of action which is interaction with the estrogenic or TH signalling pathways. These results suggests that the classical way of studying endocrine disruptors, that is by defining precise "end points" based on classical risk assessment data can some time be misleading because the focus on a specific type of defect [62,72]. In these cases, phenotypes based on unknown mode of action of the molecule will be overlooked. We believe that zebrafish and *Xenopus*, that allow medium-throughput screening of EDCs, will be powerful systems to test the effects of these compounds in an unbiased manner.

## List of Abbreviations

BPA: bisphenol A; BPC: bisphenol C; BPE: bisphenol E; BPF: bisphenol F; hpf: hours post ferilization; ER: estrogen receptor; TR: thyroid hormone receptor

## Competing interests

The authors declare they have no competing interests.

## Authors' contributions

YG, SSM, JBF, LB, DZ, JPC, PB and MAL performed experiments. YG, DZ, JPC, PB, BD and VL analysed the data. YG, BD and VL wrote the paper. All authors read and approved the final manuscript.

## Supplementary Material

Additional file 1 **Metabolic profile of BPA and BPF**. (A) Typical radio-HPLC metabolic profile obtained following incubation of radio-labeled BPA (70 μM [^14^C]-BPA) in a volume of 25 ml of E3 1X medium in the presence of 10 zebrafish embryos for 72h. Only a peak of BPA and unexcretable metabolic products are found in the water. It is noteworthy that 99.8% of the radioactivity is found in the water 24h later, indicating that only 0.2% of the BPA in the aquarium water is taken up by the embryos. (B) Typical HPLC profile of radiochemicals present in zebrafish embryos (left) and in E3 medium (right), exposed to ^3^H-BPF (5.10^-5^M). Analyses were performed on samples collected at the end of the experiment (72h exposure). Click here for file

Additional file 2 **Early BPA treatment leads to a full BPA phenotype at 50 hpf**. (A) Control untreated embryos. (B) BPA treatment from 6-10 hpf or (C) 10-15 hpf lead to a full penetrence of the otolith aggregate induced phenotype. All embryos were photographed at 50 hpf. Click here for file

Additional file 3 **Expression of markers of inner ear development in zebrafish embryos that are not affected by BPA treatment**. (A,B) *egr2a *expression at 18 hpf is unaffected under BPA treatment. (C-F) Expression of anterior markers of the otic vesicle (arrows), *athonal1 (atoh1a) *and *hmx3 *at 26 hpf, are not affected by BPA exposure. (G,H) Expression of a dorsal marker of the otic vesicle, *dlx3b *at 26 hpf, remains unaffected by BPA treatment. (I,J) Expression of *msxC *at 50 hpf in the anterior (ac) lateral (lc) and posterior cristae (pc) remains unaffected after BPA treatment. Click here for file

Additional file 4 ***oc90 *expression in the otic vesicle is up-regulated after BPE treatment**. (A) Control embryo showing normal expression of *oc90 *in the otic vesicle at 24 hpf. (B) BPE treated embryo treated from 6 hpf onwards, fixed at 24 hpf showing up-regulation of *oc90 *in the otic vesicle). Click here for file

Additional file 5 **Loss of *oc90 *does not abolish the BPA induced phenotype**. (A) Control otolith. (B) BPA treated embryos form 6 hpf onwards showing otolith aggregates, (black arrow) in both otoliths. (C) *oc90 *morpholino injected embryo with an absent anterior otolith. (D) *oc90 *morpholino injected embryo treated with BPA form 6 hpf onwards showing an aggregate of otolith (black arrow) in the only developing otolith. All embryos were observed at 50 hpf. Click here for file

Additional file 6 **BPA is a weak agonist for the three zebrafish ERs**. (A) Limited proteolysis assays of *in vitro *translated zebrafish Erα, Erβ-A and Erβ-B with E2 as ligand (right hand blots, arrows) and BPA as ligand (left hand blots, arrows). For each proteolysis panel, the first lane represents the undigested protein, lane 2 shows digestion of the receptor in the absence of ligand, lanes 3 to 8 show increased digestion of the receptor in the presence of 10 fold decreasing concentrations of E2 and BPA from 10^-3^M to 10^-8 ^M. (B) BPA weakly activates transcriptional responses with each of the zebrafish ERs in vitro. Transient co-transfections of zebrafish esr1, esr2a and esr2b and ERE-luciferase were carried out in Hela cells. Following transfection cells were treated with increasing doses of either BPA (left graphs) or E2 (right graphs). Note that E2 activates transcription from each ZfER with an ED 50 of around 1 nM, whereas the ED50s for BPA are 1 μM. Click here for file

Additional file 7 **The BPA phenotype is observed in low dose alpha1a1 MO**. (A) Control embryo with two otoliths. (B) BPA treated embryo (70 μM, form 6-50 hpf) showing otolith aggregates, (black arrow, the anterior otolith aggregates are in focus). (C) Low dose of alpha1a1 MO leads to the development of one small otolith (white arrow). (D) Low dose of alpha1a1 MO injected embryo treated with BPA (70 μM, form 6-50 hpf) lead to small otoliths formation. Strong otolith aggregates as seen in B were never observed in the low dose of alpha1a1 MO injected embryo treated with BPA, however a mild aggregate (4-5 otoliths) as shown in D (arrow) can be observed. All embryos were observed at 50 hpf. Click here for file

## References

[B1] Staples CA Davis JW An examination of the physical properties, fate, ecotoxicity and potential environmental risks for a series of propylene glycol ethers Chemosphere 2002 49 1 61 73 10.1016/S0045-6535(02)00176-5 12243331

[B2] Chapin RE Adams J Boekelheide K Gray LE Jr Hayward SW Lees PS McIntyre BS Portier KM Schnorr TM Selevan SG NTP-CERHR expert panel report on the reproductive and developmental toxicity of bisphenol A Birth Defects Res B Dev Reprod Toxicol 2008 83 3 157 395 10.1002/bdrb.20147 18613034

[B3] Focazio MJ Kolpin DW Barnes KK Furlong ET Meyer MT Zaugg SD Barber LB Thurman ME A national reconnaissance for pharmaceuticals and other organic wastewater contaminants in the United States--II) untreated drinking water sources Sci Total Environ 2008 402 2-3 201 216 10.1016/j.scitotenv.2008.02.021 18433838

[B4] Belfroid A van Velzen M van der Horst B Vethaak D Occurrence of bisphenol A in surface water and uptake in fish: evaluation of field measurements Chemosphere 2002 49 1 97 103 10.1016/S0045-6535(02)00157-1 12243336

[B5] Kolpin DW Furlong ET Meyer MT Thurman EM Zaugg SD Barber LB Buxton HT Pharmaceuticals, hormones, and other organic wastewater contaminants in U.S. streams, 1999-2000: a national reconnaissance Environ Sci Technol 2002 36 6 1202 1211 10.1021/es011055j 11944670

[B6] Kuch HM Ballschmiter K Determination of endocrine-disrupting phenolic compounds and estrogens in surface and drinking water by HRGC-(NCI)-MS in the picogram per liter range Environ Sci Technol 2001 35 15 3201 3206 10.1021/es010034m 11506003

[B7] Basheer C Lee HK Tan KS Endocrine disrupting alkylphenols and bisphenol-A in coastal waters and supermarket seafood from Singapore Mar Pollut Bull 2004 48 11-12 1161 1167 10.1016/j.marpolbul.2004.04.009 15172824

[B8] Calafat AM Kuklenyik Z Reidy JA Caudill SP Ekong J Needham LL Urinary concentrations of bisphenol A and 4-nonylphenol in a human reference population Environ Health Perspect 2005 113 4 391 395 10.1289/ehp.7534 15811827 PMC1278476

[B9] Takeuchi T Tsutsumi O Serum bisphenol a concentrations showed gender differences, possibly linked to androgen levels Biochem Biophys Res Commun 2002 291 1 76 78 10.1006/bbrc.2002.6407 11829464

[B10] Ikezuki Y Tsutsumi O Takai Y Kamei Y Taketani Y Determination of bisphenol A concentrations in human biological fluids reveals significant early prenatal exposure Hum Reprod 2002 17 11 2839 2841 10.1093/humrep/17.11.2839 12407035

[B11] Schonfelder G Flick B Mayr E Talsness C Paul M Chahoud I In utero exposure to low doses of bisphenol A lead to long-term deleterious effects in the vagina Neoplasia 2002 4 2 98 102 10.1038/sj.neo.7900212 11896564 PMC1550317

[B12] Sun Y Irie M Kishikawa N Wada M Kuroda N Nakashima K Determination of bisphenol A in human breast milk by HPLC with column-switching and fluorescence detection Biomed Chromatogr 2004 18 8 501 507 10.1002/bmc.345 15386523

[B13] Chen MY Ike M Fujita M Acute toxicity, mutagenicity, and estrogenicity of bisphenol-A and other bisphenols Environ Toxicol 2002 17 1 80 86 10.1002/tox.10035 11847978

[B14] Perez P Pulgar R Olea-Serrano F Villalobos M Rivas A Metzler M Pedraza V Olea N The estrogenicity of bisphenol A-related diphenylalkanes with various substituents at the central carbon and the hydroxy groups Environ Health Perspect 1998 106 3 167 174 9449681 10.1289/ehp.98106167 PMC1533034

[B15] Satoh K Ohyama K Aoki N Iida M Nagai F Study on anti-androgenic effects of bisphenol a diglycidyl ether (BADGE), bisphenol F diglycidyl ether (BFDGE) and their derivatives using cells stably transfected with human androgen receptor, AR-EcoScreen Food Chem Toxicol 2004 42 6 983 993 10.1016/j.fct.2004.02.011 15110108

[B16] Waring RH Harris RM Endocrine disrupters: a human risk? Mol Cell Endocrinol 2005 244 1-2 2 9 10.1016/j.mce.2005.02.007 16271281

[B17] Crain DA Eriksen M Iguchi T Jobling S Laufer H LeBlanc GA Guillette LJ Jr An ecological assessment of bisphenol-A: evidence from comparative biology Reprod Toxicol 2007 24 2 225 239 10.1016/j.reprotox.2007.05.008 17604601

[B18] Maffini MV Rubin BS Sonnenschein C Soto AM Endocrine disruptors and reproductive health: the case of bisphenol-A Mol Cell Endocrinol 2006 254-255 179 186 10.1016/j.mce.2006.04.033 16781053

[B19] Wetherill YB Akingbemi BT Kanno J McLachlan JA Nadal A Sonnenschein C Watson CS Zoeller RT Belcher SM In vitro molecular mechanisms of bisphenol A action Reprod Toxicol 2007 24 2 178 198 10.1016/j.reprotox.2007.05.010 17628395

[B20] Kuiper GG Shughrue PJ Merchenthaler I Gustafsson JA The estrogen receptor beta subtype: a novel mediator of estrogen action in neuroendocrine systems Front Neuroendocrinol 1998 19 4 253 286 10.1006/frne.1998.0170 9799586

[B21] Pennie WD Aldridge TC Brooks AN Differential activation by xenoestrogens of ER alpha and ER beta when linked to different response elements J Endocrinol 1998 158 3 R11 14 10.1677/joe.0.158R011 9846177

[B22] Bjornstrom L Sjoberg M Mechanisms of estrogen receptor signaling: convergence of genomic and nongenomic actions on target genes Mol Endocrinol 2005 19 4 833 842 10.1210/me.2004-0486 15695368

[B23] Hiroi H Tsutsumi O Momoeda M Takai Y Osuga Y Taketani Y Differential interactions of bisphenol A and 17beta-estradiol with estrogen receptor alpha (ERalpha) and ERbeta Endocr J 1999 46 6 773 778 10.1507/endocrj.46.773 10724352

[B24] Richter CA Taylor JA Ruhlen RL Welshons WV Vom Saal FS Estradiol and Bisphenol A stimulate androgen receptor and estrogen receptor gene expression in fetal mouse prostate mesenchyme cells Environ Health Perspect 2007 115 6 902 908 10.1289/ehp.9804 17589598 PMC1892114

[B25] Wozniak AL Bulayeva NN Watson CS Xenoestrogens at picomolar to nanomolar concentrations trigger membrane estrogen receptor-alpha-mediated Ca2+ fluxes and prolactin release in GH3/B6 pituitary tumor cells Environ Health Perspect 2005 113 4 431 439 10.1289/ehp.7505 15811834 PMC1278483

[B26] Zsarnovszky A Le HH Wang HS Belcher SM Ontogeny of rapid estrogen-mediated extracellular signal-regulated kinase signaling in the rat cerebellar cortex: potent nongenomic agonist and endocrine disrupting activity of the xenoestrogen bisphenol A Endocrinology 2005 146 12 5388 5396 10.1210/en.2005-0565 16123166

[B27] Moriyama K Tagami T Akamizu T Usui T Saijo M Kanamoto N Hataya Y Shimatsu A Kuzuya H Nakao K Thyroid hormone action is disrupted by bisphenol A as an antagonist J Clin Endocrinol Metab 2002 87 11 5185 5190 10.1210/jc.2002-020209 12414890

[B28] Zoeller RT Environmental chemicals as thyroid hormone analogues: new studies indicate that thyroid hormone receptors are targets of industrial chemicals? Mol Cell Endocrinol 2005 242 1-2 10 15 10.1016/j.mce.2005.07.006 16150534

[B29] Zoeller RT Bansal R Parris C Bisphenol-A, an environmental contaminant that acts as a thyroid hormone receptor antagonist in vitro, increases serum thyroxine, and alters RC3/neurogranin expression in the developing rat brain Endocrinology 2005 146 2 607 612 10.1210/en.2004-1018 15498886

[B30] Iwamuro S Yamada M Kato M Kikuyama S Effects of bisphenol A on thyroid hormone-dependent up-regulation of thyroid hormone receptor alpha and beta and down-regulation of retinoid X receptor gamma in *Xenopus *tail culture Life Sci 2006 79 23 2165 2171 10.1016/j.lfs.2006.07.013 16905155

[B31] Fini JB Le Mevel S Turque N Palmier K Zalko D Cravedi JP Demeneix BA An in vivo multiwell-based fluorescent screen for monitoring vertebrate thyroid hormone disruption Environ Sci Technol 2007 41 16 5908 5914 10.1021/es0704129 17874805

[B32] Duan Z Zhu L Zhu L Kun Y Zhu X Individual and joint toxic effects of pentachlorophenol and bisphenol A on the development of zebrafish (Danio rerio) embryo Ecotoxicol Environ Saf 2008 71 3 774 780 10.1016/j.ecoenv.2008.01.021 18359083

[B33] Pastva SD Villalobos SA Kannan K Giesy JP Morphological effects of Bisphenol-A on the early life stages of medaka (Oryzias latipes) Chemosphere 2001 45 4-5 535 541 10.1016/S0045-6535(01)00018-2 11680749

[B34] Baba K Okada K Kinoshita T Imaoka S Bisphenol A disrupts Notch signaling by inhibiting gamma-secretase activity and causes eye dysplasia of *Xenopus laevis* Toxicol Sci 2009 108 2 344 355 10.1093/toxsci/kfp025 19218331

[B35] Heimeier RA Das B Buchholz DR Shi YB The xenoestrogen bisphenol A inhibits postembryonic vertebrate development by antagonizing gene regulation by thyroid hormone Endocrinology 2009 150 6 2964 2973 10.1210/en.2008-1503 19228888 PMC2689811

[B36] Imaoka S Mori T Kinoshita T Bisphenol A causes malformation of the head region in embryos of *Xenopus laevis *and decreases the expression of the ESR-1 gene mediated by Notch signaling Biol Pharm Bull 2007 30 2 371 374 10.1248/bpb.30.371 17268083

[B37] Kimmel CB Ballard WW Kimmel SR Ullmann B Schilling TF Stages of embryonic development of the zebrafish Dev Dyn 1995 203 3 253 310 8589427 10.1002/aja.1002030302

[B38] Zalko D Soto AM Dolo L Dorio C Rathahao E Debrauwer L Faure R Cravedi JP Biotransformations of bisphenol A in a mammalian model: answers and new questions raised by low-dose metabolic fate studies in pregnant CD1 mice Environ Health Perspect 2003 111 3 309 319 10.1289/ehp.5603 12611660 PMC1241388

[B39] Cabaton N Zalko D Rathahao E Canlet C Delous G Chagnon MC Cravedi JP Perdu E Biotransformation of bisphenol F by human and rat liver subcellular fractions Toxicol In Vitro 2008 22 7 1697 1704 10.1016/j.tiv.2008.07.004 18672047

[B40] Nieuwkoop PD Faber J Normal table of *Xenopus laevis* 1994 Garland Publishing Inc, New York Daudin

[B41] Thisse C Thisse B Schilling TF Postlethwait JH Structure of the zebrafish snail1 gene and its expression in wild-type, spadetail and no tail mutant embryos Development 1993 119 4 1203 1215 8306883 10.1242/dev.119.4.1203

[B42] Avanesov A Dahm R Sewell WF Malicki JJ Mutations that affect the survival of selected amacrine cell subpopulations define a new class of genetic defects in the vertebrate retina Dev Biol 2005 285 1 138 155 10.1016/j.ydbio.2005.06.009 16231865

[B43] Escriva H Bertrand S Germain P Robinson-Rechavi M Umbhauer M Cartry J Duffraisse M Holland L Gronemeyer H Laudet V Neofunctionalization in vertebrates: the example of retinoic acid receptors PLoS Genet 2006 2 7 e102 10.1371/journal.pgen.0020102 16839186 PMC1500811

[B44] Legler J Zeinstra LM Schuitemaker F Lanser PH Bogerd J Brouwer A Vethaak AD De Voogt P Murk AJ Van der Burg B Comparison of in vivo and in vitro reporter gene assays for short-term screening of estrogenic activity Environ Sci Technol 2002 36 20 4410 4415 10.1021/es010323a 12387416

[B45] Hughes I Thalmann I Thalmann R Ornitz DM Mixing model systems: using zebrafish and mouse inner ear mutants and other organ systems to unravel the mystery of otoconial development Brain Res 2006 1091 1 58 74 10.1016/j.brainres.2006.01.074 16529728 PMC2100415

[B46] Ramakrishnan S Wayne NL Impact of bisphenol-A on early embryonic development and reproductive maturation Reprod Toxicol 2008 25 2 177 183 10.1016/j.reprotox.2007.11.002 18191535

[B47] Whitfield TT Granato M van Eeden FJ Schach U Brand M Furutani-Seiki M Haffter P Hammerschmidt M Heisenberg CP Jiang YJ Mutations affecting development of the zebrafish inner ear and lateral line Development 1996 123 241 254 9007244 10.1242/dev.123.1.241

[B48] Riley BB Zhu C Janetopoulos C Aufderheide KJ A critical period of ear development controlled by distinct populations of ciliated cells in the zebrafish Dev Biol 1997 191 2 191 201 10.1006/dbio.1997.8736 9398434

[B49] Colantonio JR Vermot J Wu D Langenbacher AD Fraser S Chen JN Hill KL The dynein regulatory complex is required for ciliary motility and otolith biogenesis in the inner ear Nature 2009 457 7226 205 209 10.1038/nature07520 19043402 PMC3821763

[B50] Hans S Westerfield M Changes in retinoic acid signaling alter otic patterning Development 2007 134 13 2449 2458 10.1242/dev.000448 17522161

[B51] Solomon KS Kwak SJ Fritz A Genetic interactions underlying otic placode induction and formation Dev Dyn 2004 230 3 419 433 10.1002/dvdy.20067 15188428

[B52] Hammond KL Loynes HE Folarin AA Smith J Whitfield TT Hedgehog signalling is required for correct anteroposterior patterning of the zebrafish otic vesicle Development 2003 130 7 1403 1417 10.1242/dev.00360 12588855

[B53] Petko JA Millimaki BB Canfield VA Riley BB Levenson R Otoc1: a novel otoconin-90 ortholog required for otolith mineralization in zebrafish Dev Neurobiol 2008 68 2 209 222 10.1002/dneu.20587 18000829 PMC2730775

[B54] Blasiole B Canfield VA Vollrath MA Huss D Mohideen MA Dickman JD Cheng KC Fekete DM Levenson R Separate Na,K-ATPase genes are required for otolith formation and semicircular canal development in zebrafish Dev Biol 2006 294 1 148 160 10.1016/j.ydbio.2006.02.034 16566913

[B55] Busch-Nentwich E Sollner C Roehl H Nicolson T The deafness gene dfna5 is crucial for ugdh expression and HA production in the developing ear in zebrafish Development 2004 131 4 943 951 10.1242/dev.00961 14736743

[B56] Pittlik S Domingues S Meyer A Begemann G Expression of zebrafish aldh1a3 (raldh3) and absence of aldh1a1 in teleosts Gene Expr Patterns 2008 8 3 141 147 10.1016/j.gep.2007.11.003 18178530

[B57] Meltser I Tahera Y Simpson E Hultcrantz M Charitidi K Gustafsson JA Canlon B Estrogen receptor beta protects against acoustic trauma in mice J Clin Invest 2008 118 4 1563 1570 10.1172/JCI32796 18317592 PMC2260908

[B58] Sisneros JA Forlano PM Deitcher DL Bass AH Steroid-dependent auditory plasticity leads to adaptive coupling of sender and receiver Science 2004 305 5682 404 407 10.1126/science.1097218 15256672

[B59] Bardet PL Horard B Robinson-Rechavi M Laudet V Vanacker JM Characterization of oestrogen receptors in zebrafish (Danio rerio) J Mol Endocrinol 2002 28 3 153 163 10.1677/jme.0.0280153 12063182

[B60] Menuet A Pellegrini E Anglade I Blaise O Laudet V Kah O Pakdel F Molecular characterization of three estrogen receptor forms in zebrafish: binding characteristics, transactivation properties, and tissue distributions Biol Reprod 2002 66 6 1881 1892 10.1095/biolreprod66.6.1881 12021076

[B61] Bertrand S Thisse B Tavares R Sachs L Chaumot A Bardet PL Escriva H Duffraisse M Marchand O Safi R Unexpected novel relational links uncovered by extensive developmental profiling of nuclear receptor expression PLoS Genet 2007 3 11 e188 10.1371/journal.pgen.0030188 17997606 PMC2065881

[B62] Sassi-Messai S Gibert Y Bernard L Nishio S Ferri Lagneau KF Molina J Andersson-Lendahl M Benoit G Balaguer P Laudet V The phytoestrogen genistein affects zebrafish development through two different pathways PLoS ONE 2009 4 3 e4935 10.1371/journal.pone.0004935 19319186 PMC2655710

[B63] Abel ED Boers ME Pazos-Moura C Moura E Kaulbach H Zakaria M Lowell B Radovick S Liberman MC Wondisford F Divergent roles for thyroid hormone receptor beta isoforms in the endocrine axis and auditory system J Clin Invest 1999 104 3 291 300 10.1172/JCI6397 10430610 PMC408418

[B64] Rusch A Erway LC Oliver D Vennstrom B Forrest D Thyroid hormone receptor beta-dependent expression of a potassium conductance in inner hair cells at the onset of hearing Proc Natl Acad Sci USA 1998 95 26 15758 15762 10.1073/pnas.95.26.15758 9861043 PMC28117

[B65] Morvan Dubois G Sebillot A Kuiper GG Verhoelst CH Darras VM Visser TJ Demeneix BA Deiodinase activity is present in *Xenopus laevis *during early embryogenesis Endocrinology 2006 147 10 4941 4949 10.1210/en.2006-0609 16825318

[B66] Blasiole B Degrave A Canfield V Boehmler W Thisse C Thisse B Mohideen MA Levenson R Differential expression of Na,K-ATPase alpha and beta subunit genes in the developing zebrafish inner ear Dev Dyn 2003 228 3 386 392 10.1002/dvdy.10391 14579377

[B67] Haddon C Lewis J Early ear development in the embryo of the zebrafish, Danio rerio J Comp Neurol 1996 365 1 113 128 10.1002/(SICI)1096-9861(19960129)365:1<113::AID-CNE9>3.0.CO;2-6 8821445

[B68] Nicolson T The genetics of hearing and balance in zebrafish Annu Rev Genet 2005 39 9 22 10.1146/annurev.genet.39.073003.105049 16285850

[B69] Hawkins RD Bashiardes S Powder KE Sajan SA Bhonagiri V Alvarado DM Speck J Warchol ME Lovett M Large scale gene expression profiles of regenerating inner ear sensory epithelia PLoS ONE 2007 2 6 e525 10.1371/journal.pone.0000525 17565378 PMC1888727

[B70] Kausch U Alberti M Haindl S Budczies J Hock B Biomarkers for exposure to estrogenic compounds: gene expression analysis in zebrafish (Danio rerio) Environ Toxicol 2008 23 1 15 24 10.1002/tox.20306 18214933

[B71] Thalmann R Ignatova E Kachar B Ornitz DM Thalmann I Development and maintenance of otoconia: biochemical considerations Ann N Y Acad Sci 2001 942 162 178 10.1111/j.1749-6632.2001.tb03743.x 11710459

[B72] Markov GV Paris M Bertrand S Laudet V The evolution of the ligand/receptor couple: A long road from comparative endocrinology to comparative genomics Mol Cell Endocrinol 2008 293 1-2 5 16 10.1016/j.mce.2008.06.011 18634845

[B73] Bever MM Jean YY Fekete DM Three-dimensional morphology of inner ear development in Xenopus laevis Dev Dyn 2003 227 3 422 430 10.1002/dvdy.10316 12815629

