# Supplementary figures and images for: Bisphenol A induces otolith malformations during vertebrate embryogenesis
Source: BMC Dev Biol. 2011 Jan 26;11:4. doi: 10.1186/1471-213X-11-4 (PMC3040707; doi:10.1186/1471-213X-11-4)

## Slide 1
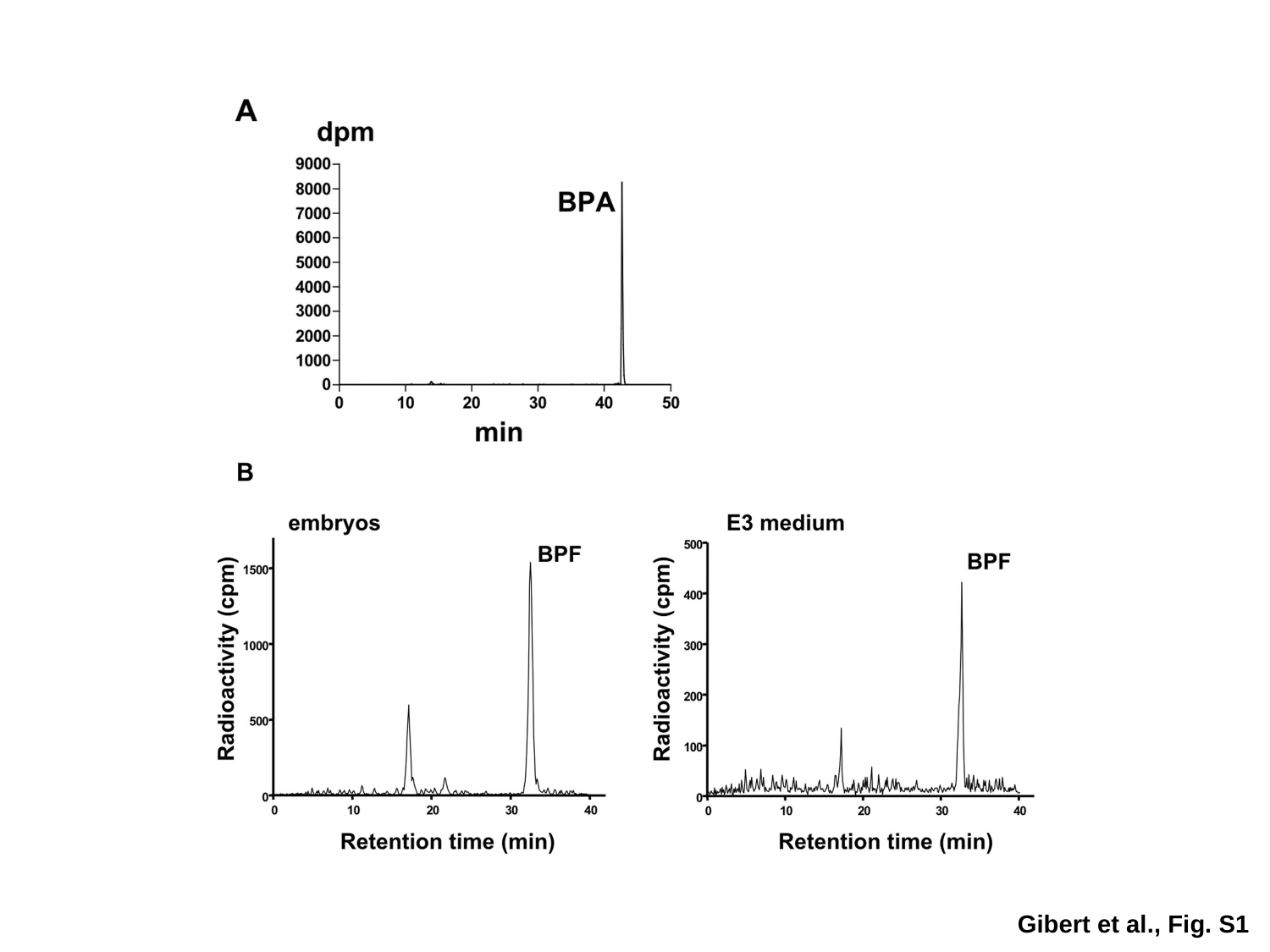

Gibert et al., Fig. S1

Supplement: Additional file 1 — Metabolic profile of BPA and BPF. (A) Typical radio-HPLC metabolic profile obtained following incubation of radio-labeled BPA (70 μM [14C]-BPA) in a volume of 25 ml of E3 1X medium in the presence of 10 zebrafish embryos for 72h. Only a peak of BPA and unexcretable metabolic products are found in the water. It is noteworthy that 99.8% of the radioactivity is found in the water 24h later, indicating that only 0.2% of the BPA in the aquarium water is taken up by the embryos. (B) Typical HPLC profile of radiochemicals present in zebrafish embryos (left) and in E3 medium (right), exposed to 3H-BPF (5.10-5M). Analyses were performed on samples collected at the end of the experiment (72h exposure). [file 1471-213X-11-4-S1.PPT]

## Slide 1
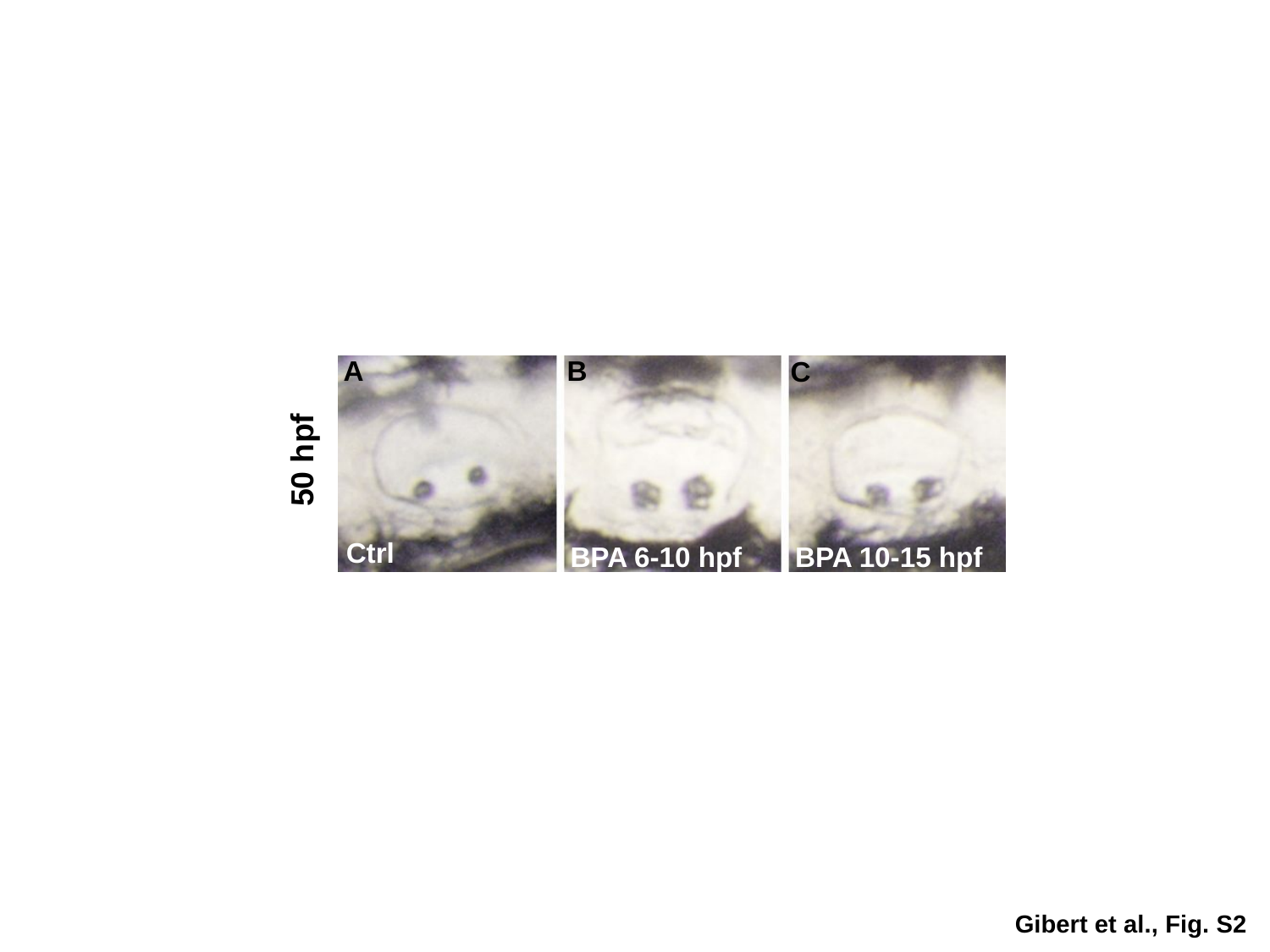

A
B
C
50 hpf
Ctrl
BPA 6-10 hpf
BPA 10-15 hpf
Gibert et al., Fig. S2

Supplement: Additional file 2 — Early BPA treatment leads to a full BPA phenotype at 50 hpf. (A) Control untreated embryos. (B) BPA treatment from 6-10 hpf or (C) 10-15 hpf lead to a full penetrence of the otolith aggregate induced phenotype. All embryos were photographed at 50 hpf. [file 1471-213X-11-4-S2.PPT]

## Slide 1
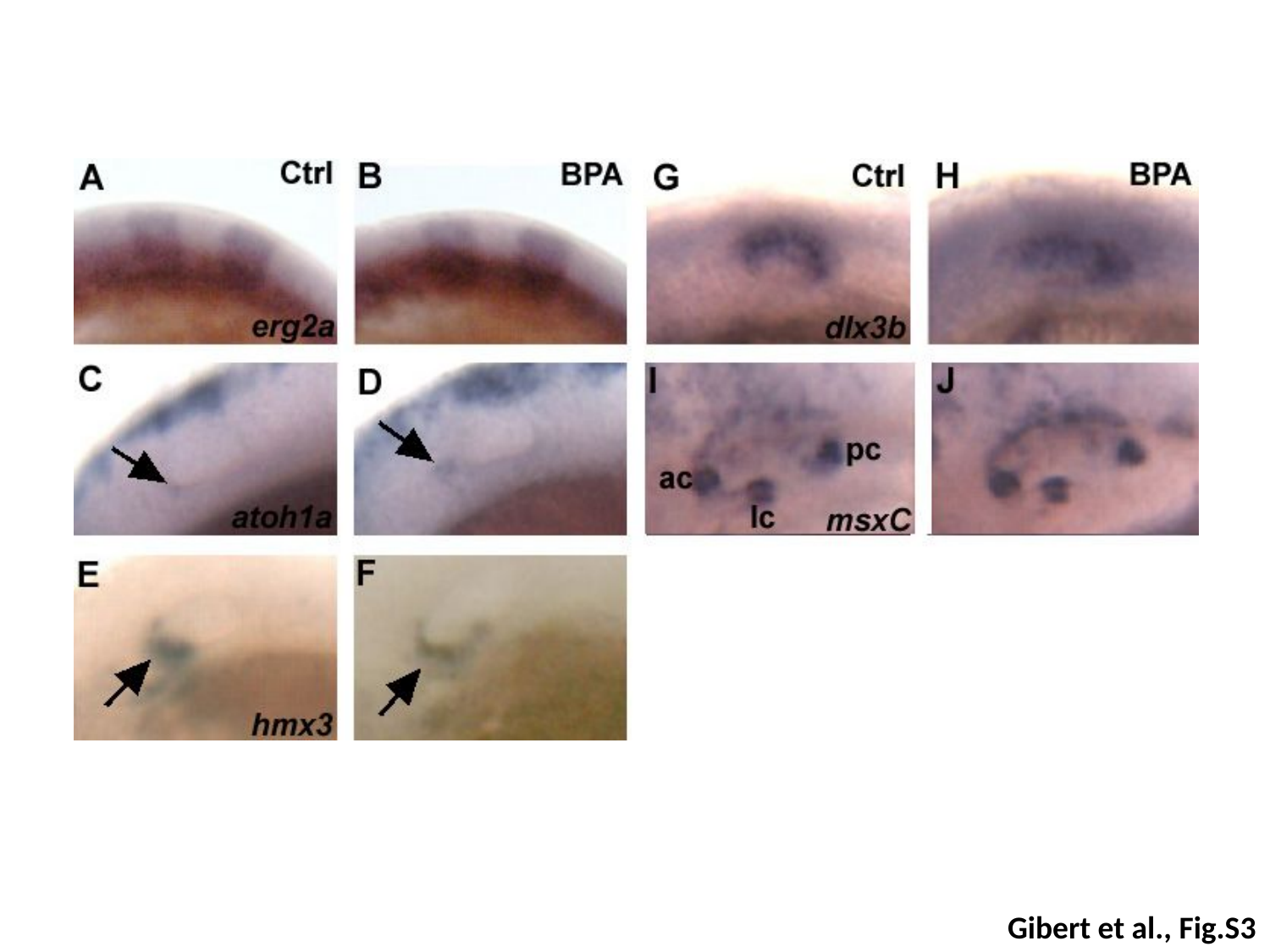

Gibert et al., Fig.S3

Supplement: Additional file 3 — Expression of markers of inner ear development in zebrafish embryos that are not affected by BPA treatment. (A,B) egr2a expression at 18 hpf is unaffected under BPA treatment. (C-F) Expression of anterior markers of the otic vesicle (arrows), athonal1 (atoh1a) and hmx3 at 26 hpf, are not affected by BPA exposure. (G,H) Expression of a dorsal marker of the otic vesicle, dlx3b at 26 hpf, remains unaffected by BPA treatment. (I,J) Expression of msxC at 50 hpf in the anterior (ac) lateral (lc) and posterior cristae (pc) remains unaffected after BPA treatment. [file 1471-213X-11-4-S3.PPT]

## Slide 1
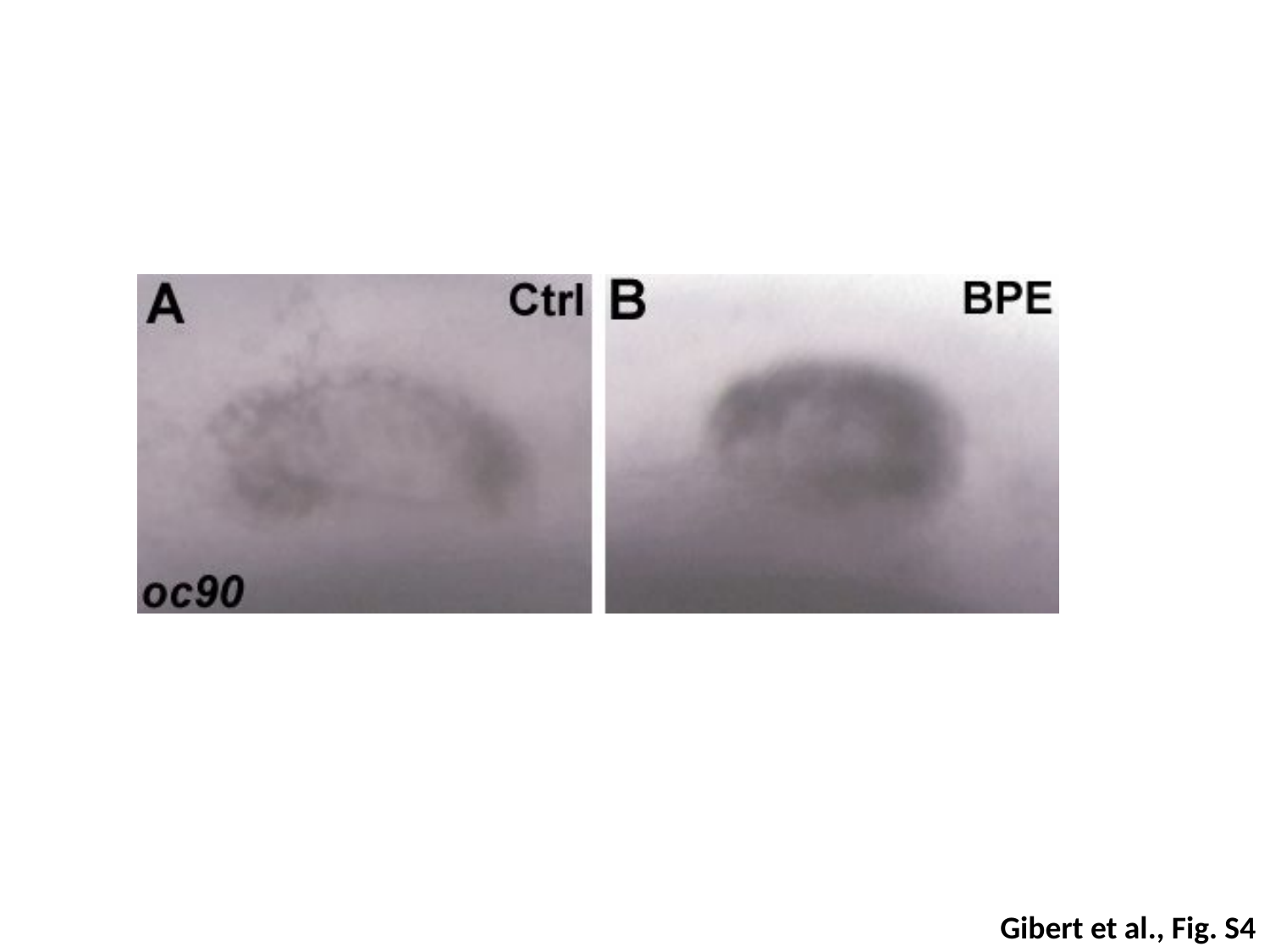

Gibert et al., Fig. S4

Supplement: Additional file 4 — oc90 expression in the otic vesicle is up-regulated after BPE treatment. (A) Control embryo showing normal expression of oc90 in the otic vesicle at 24 hpf. (B) BPE treated embryo treated from 6 hpf onwards, fixed at 24 hpf showing up-regulation of oc90 in the otic vesicle). [file 1471-213X-11-4-S4.PPT]

## Slide 1
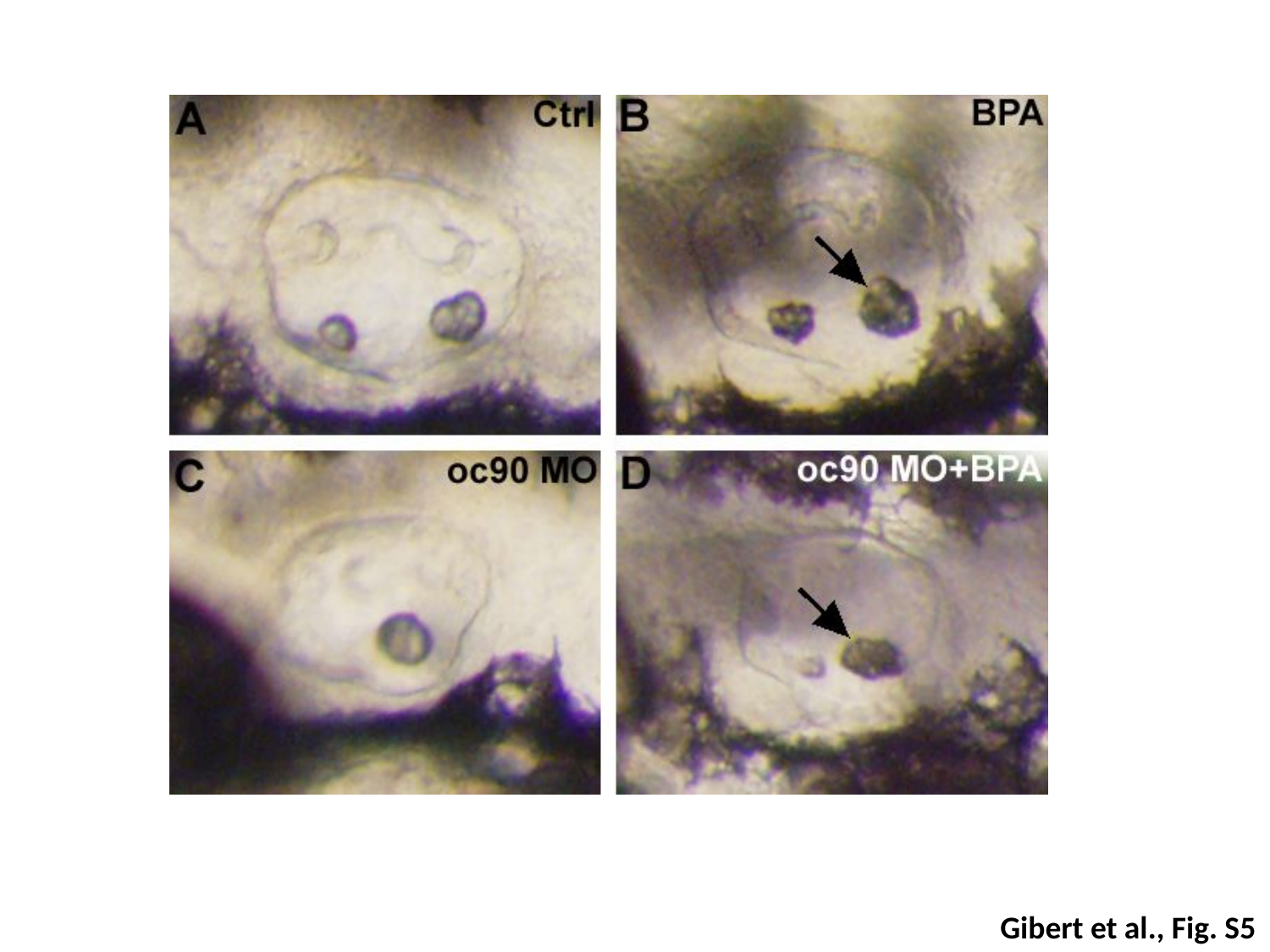

Gibert et al., Fig. S5

Supplement: Additional file 5 — Loss of oc90 does not abolish the BPA induced phenotype. (A) Control otolith. (B) BPA treated embryos form 6 hpf onwards showing otolith aggregates, (black arrow) in both otoliths. (C) oc90 morpholino injected embryo with an absent anterior otolith. (D) oc90 morpholino injected embryo treated with BPA form 6 hpf onwards showing an aggregate of otolith (black arrow) in the only developing otolith. All embryos were observed at 50 hpf. [file 1471-213X-11-4-S5.PPT]

## Slide 1
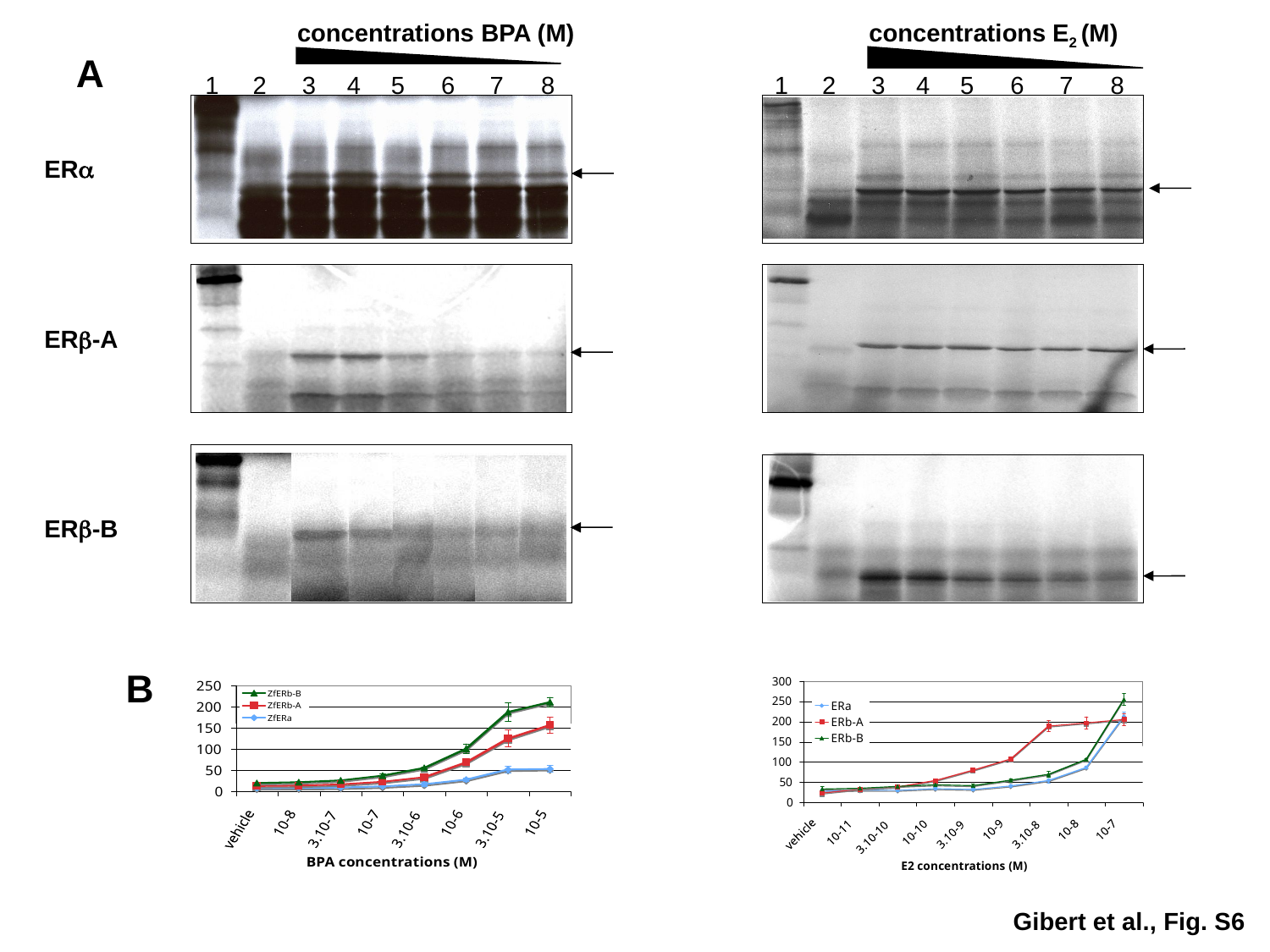

concentrations BPA (M)
concentrations E2 (M)
A
1
2
3
4
5
6
7
8
1
2
3
4
5
6
7
8
| |
| --- |
| |
| --- |
ERα
| |
| --- |
| |
| --- |
ERβ-A
| |
| --- |
| |
| --- |
ERβ-B
B
Gibert et al., Fig. S6

Supplement: Additional file 6 — BPA is a weak agonist for the three zebrafish ERs. (A) Limited proteolysis assays of in vitro translated zebrafish Erα, Erβ-A and Erβ-B with E2 as ligand (right hand blots, arrows) and BPA as ligand (left hand blots, arrows). For each proteolysis panel, the first lane represents the undigested protein, lane 2 shows digestion of the receptor in the absence of ligand, lanes 3 to 8 show increased digestion of the receptor in the presence of 10 fold decreasing concentrations of E2 and BPA from 10-3M to 10-8 M. (B) BPA weakly activates transcriptional responses with each of the zebrafish ERs in vitro. Transient co-transfections of zebrafish esr1, esr2a and esr2b and ERE-luciferase were carried out in Hela cells. Following transfection cells were treated with increasing doses of either BPA (left graphs) or E2 (right graphs). Note that E2 activates transcription from each ZfER with an ED 50 of around 1 nM, whereas the ED50s for BPA are 1 μM. [file 1471-213X-11-4-S6.PPT]

## Slide 1
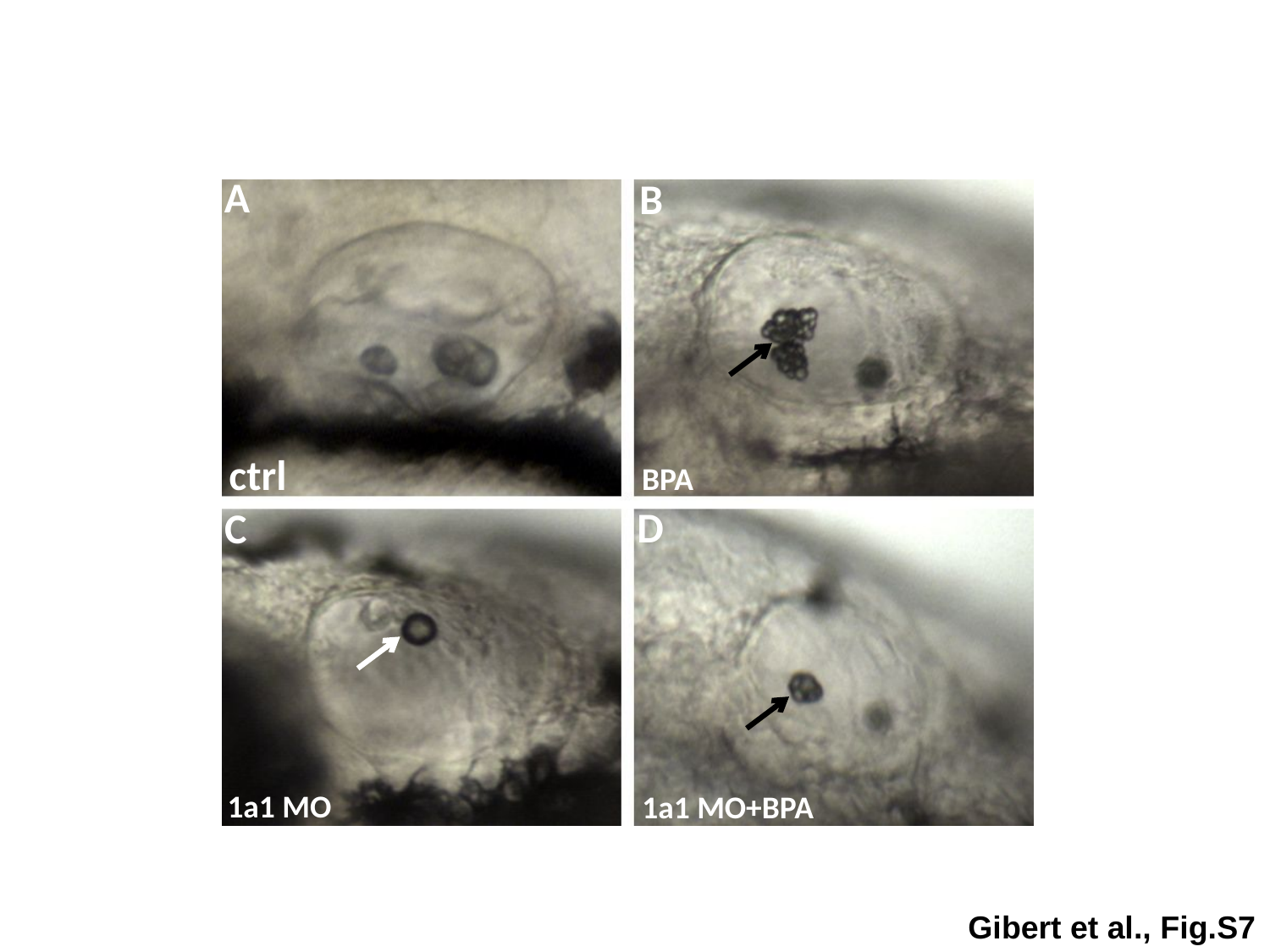

A
B
ctrl
BPA
D
C
1a1 MO
1a1 MO+BPA
Gibert et al., Fig.S7

Supplement: Additional file 7 — The BPA phenotype is observed in low dose alpha1a1 MO. (A) Control embryo with two otoliths. (B) BPA treated embryo (70 μM, form 6-50 hpf) showing otolith aggregates, (black arrow, the anterior otolith aggregates are in focus). (C) Low dose of alpha1a1 MO leads to the development of one small otolith (white arrow). (D) Low dose of alpha1a1 MO injected embryo treated with BPA (70 μM, form 6-50 hpf) lead to small otoliths formation. Strong otolith aggregates as seen in B were never observed in the low dose of alpha1a1 MO injected embryo treated with BPA, however a mild aggregate (4-5 otoliths) as shown in D (arrow) can be observed. All embryos were observed at 50 hpf. [file 1471-213X-11-4-S7.PPTX]
